# In vivo validation of highly customized cranial Ti-6AL-4V ELI prostheses fabricated through incremental forming and superplastic forming: an ovine model study

**DOI:** 10.1038/s41598-024-57629-3

**Published:** 2024-04-04

**Authors:** Silvia Brogini, Alberto Crovace, Antonio Piccininni, Giuseppe Serratore, Gregorio Marchiori, Melania Maglio, Pasquale Guglielmi, Angela Cusanno, Luigi De Napoli, Romina Conte, Milena Fini, Giuseppina Ambrogio, Gianfranco Palumbo, Gianluca Giavaresi

**Affiliations:** 1 Scienze e Tecnologie Chirurgiche, IRCCS Istituto Ortopedico Rizzoli, Via Di Barbiano, 1/10, Bologna, Italy; 2 Dipartimento di Medicina Veterinaria, Università di Sassari, Via Vienna 2, 07100 Sassari, Italy; 3 Dipartimento di Meccanica, Matematica e Management, Politecnico di Bari, Via Orabona 4, 70125 Bari, Italy; 4 Dipartimento di Ingegneria Meccanica, Energetica e Gestionale, Università Della Calabria, Ponte P. Bucci Cubo 45C, 87036 Rende, CS Italy; 5 Direzione Scientifica, IRCCS Istituto Ortopedico Rizzoli, Via di Barbiano, 1/10, Bologna, Italy

**Keywords:** Health care, Biomedical engineering, Implants

## Abstract

Cranial reconstructions are essential for restoring both function and aesthetics in patients with craniofacial deformities or traumatic injuries. Titanium prostheses have gained popularity due to their biocompatibility, strength, and corrosion resistance. The use of Superplastic Forming (SPF) and Single Point Incremental Forming (SPIF) techniques to create titanium prostheses, specifically designed for cranial reconstructions was investigated in an ovine model through microtomographic and histomorphometric analyses. The results obtained from the explanted specimens revealed significant variations in bone volume, trabecular thickness, spacing, and number across different regions of interest (VOIs or ROIs). Those regions next to the center of the cranial defect exhibited the most immature bone, characterized by higher porosity, decreased trabecular thickness, and wider trabecular spacing. Dynamic histomorphometry demonstrated differences in the mineralizing surface to bone surface ratio (MS/BS) and mineral apposition rate (MAR) depending on the timing of fluorochrome administration. A layer of connective tissue separated the prosthesis and the bone tissue. Overall, the study provided validation for the use of cranial prostheses made using SPF and SPIF techniques, offering insights into the processes of bone formation and remodeling in the implanted ovine model.

## Introduction

Traumatic skull fractures, tumor resections, decompressive craniectomy or infections are common causes of cranial disorders that may lead to craniectomy and later reconstruction^1^. After bone flap removal, cranioplasty is performed to restore the anatomical integrity of the skull, ensuring adequate biomechanical protection of the underlying brain, as well as to preserve and improve the cerebral hemodynamics and metabolism. Autologous bone grafts have traditionally been the preferred treatment of choice for cranial reconstruction. However, due to various issues with tissue suitability and their high resorption rate, their use is now being reconsidered in favor of different alloplastic materials^2^.

Biomedical engineering has brought new materials and manufacturing methods to surgeons. In craniofacial surgery, achieving favorable physiological and aesthetic outcomes is key^3^. Cranioplasty presents an ongoing challenge for surgeons and bioengineers. Additionally, minimizing surgical time and hospitalization while considering financial impact are critical objectives for craniofacial biomedical implants^4,5^. Therefore, research for better and customized biomedical solutions from a technological standpoint must not neglect these important aspects^1,6^.

Currently, standard procedures for manufacturing prostheses mainly involve using polymethyl-methacrylate (PMMA), polyetheretherketone (PEEK), bioceramics like hydroxyapatite (HA)^7–9^, and titanium (Ti) or its alloys (Ti6Al4V). However, Ti is still the most commonly used material. A systematic review and meta-analysis conducted by Zhu et al., found that Ti cranioplasty resulted in significantly fewer postoperative complications, such as hematoma, imprecise fitting, or reoperation, compared to non-metallic implants^10^.

Custom implants are the preferred solution for achieving both biocompatibility and aesthetic compatibility. This ensures excellent aesthetic results. the CAD/CAM approach, along with innovative production platforms^11^, represents the new paradigm of this century, as it facilitates and minimizes surgical procedures^12^. Skull implants are conventionally manufactured using the Computer Numerical Control (CNC) milling process^13^. However, this method has several drawbacks, including high costs, material waste, long manufacturing time, and inadequate exploration of the material’s deformation potential^14^. On the other hand, additive manufacturing (AM) has shown disruptive advantages when applied to the biomedical sector^15^: the capability to manufacture the final part layer by layer allows to achieve very complex geometry, therefore satisfying the product’s full customization^16^. Nevertheless, some limitations counterbalance to some extent the advantages: within the pre-process stage, the conversion of the scan data, the slicing of the geometry and the creation of the STL still represent a time-consuming step and dilate the production time^19,20^. From the process perspective, literature reports that some of the AM techniques lack of accuracy (EBM) or geometric limitations (WAAM), whereas other suffer from long processing time (SLS) or poor mechanical properties of the final component (FDM)^17,18^. Moreover, as an additional aspect to be accounted, AM techniques are designed and developed to process only particular category of materials, thus needing the modification of the hardware of the system in case of particular requirements^19^. In this regard, literature reports that some limitations come from the material itself: there are still particular category of metallic materials difficult to be processed: cobalt alloys, for example, are still cumbersome to manufacture whereas the adoption of Mg-based alloys is limited due to the high reactivity with oxygen especially if the raw material is supplied in powder^20–22^^.^.

When prostheses have complex profiles, undercuts, or thin walls, innovative sheet metal forming processes such as Superplastic Forming (SPF) and Single Point Incremental Forming (SPIF) can be considered viable alternatives. These processes use Ti sheet, instead of the bulk part to quickly obtain complex, patient-specific geometries^1,23–25^. Both of these technologies follow the philosophy of additive manufacturing, modeling materials based on medical needs while minimizing waste. When comparing the two manufacturing routes, each has its own balance of advantages and drawbacks. Although the SPF has the potential to achieve high levels of complexity, it requires larger initial investments for equipment^26^. As a consequence, it has been ranked as the most consumptive option^27^. In contrast, the SPIF technology is less expensive but results in lower geometrical complexity and dimensional accuracy of the final part (the springback effect are more pronounced as well as the residual state of stress that generally needs a post-forming heat treatment)^28^. Our previous research studies have tested these approaches for manufacturing patient-oriented cranial prostheses based on a clinical case. Piccininni et al.^23^ examined two different Ti alloys and geometries to guide the design of the manufacturing processes. Numerical simulations and experimental texts were performed to determine optimal conditions for deformation during the forming process and to prevent breakage during the manufacturing process. The characteristics of the prostheses were measured and compared with simulation data, finding a strong correlation between numerical and experimental results^23^. Palumbo et al.^25^ analyzed the chemical contamination and mechanical alteration of titanium alloys during superplastic forming using optical emission spectrometry, nanoindentation, and metallographic analysis techniques. Cytotoxicity tests also showed that oxygen enrichment during production has no significant impact on cell viability. Lastly, the mechanical performances were investigated by Ambrogio et al.^4^, through drop tests, revealing no failures of the prostheses, neither in the area of impact nor in the anchoring region, achieving an energy absorption of more than 70%^21^. In the wake of these promising results, the aim of this paper was to validate the in vivo use of highly customized prostheses fabricated through SPF and SPIF techniques in an ovine model. Following the same needs that are usually employed for human implants, a step by step approach was set; a virtual 3D model of the skull was obtained, and the defect region and prostheses position were defined, resulting in a CAD reconstruction of the prostheses geometry. After manufacturing, the customized Ti6Al4V (ELI) prostheses were implanted in sheep underwent craniectomy and reconstruction of the skull. The behaviour of the prostheses was assessed by means of microtomography and static and dynamic histomorphometric analysis, assuming that there might be differences in the response of the bone tissue to the two types of prostheses manufactured.

## Materials and methods

### Prostheses’ design and manufacturing

#### Design & manufacturing of the guiding mask

The experimental investigation has been driven by the fundamental goal to customize the damage and reconstruction procedure, according to the specific needs of the implants used for humans. For this reason, a robust step by step approach was set combining geometrical, surgical and manufacturing issues. First of all, each test animal was subjected to a computerized tomography (CT) scan to obtain the virtual model of the whole skull thorough a reverse engineering reconstruction.

Such a preliminary step was performed for obtaining a virtual model of the portion of the skull in which the prosthesis will have to be implanted, which is fundamental for the virtual modelling of the defect to be created. The skull geometry was then processed as an STL file in order to be manipulated by means of CAx tools^29^ in order to define the prosthesis geometry and design its manufacturing process.

**Figure 1:**
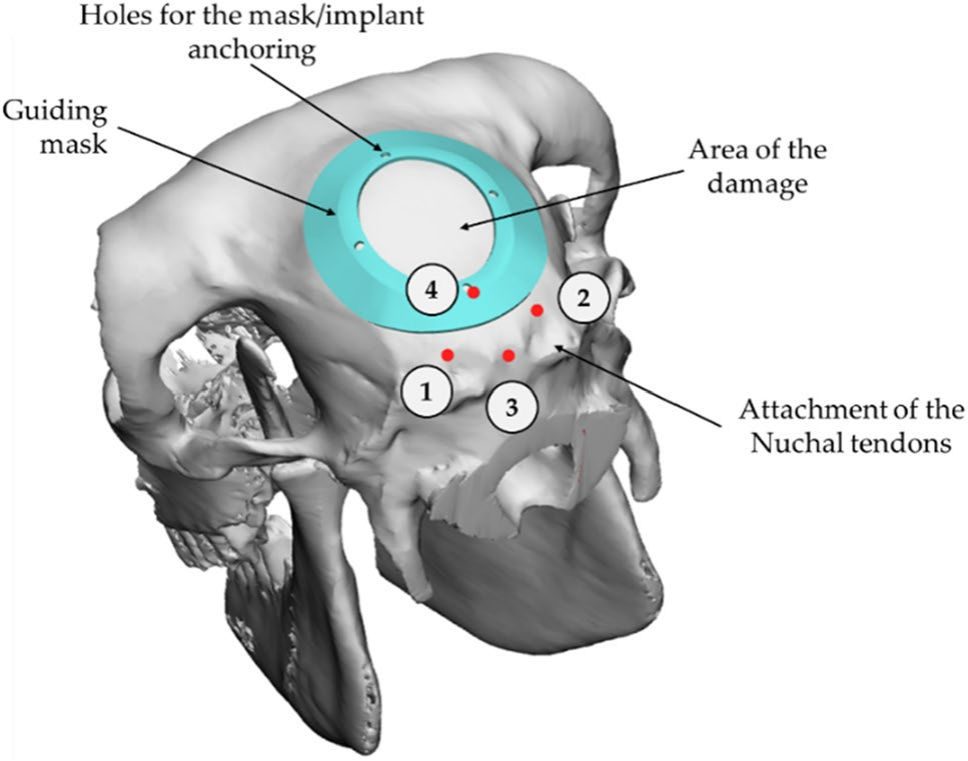
The 3D overview of the guiding mask positioning on the skull along with the reference nuchal tendons’ attachment points.

The virtual geometry reconstruction is mandatory not only for identifying the most suitable morphology of the defect and the consequent prosthetic geometry, but also for creating a customized "guide-mask" to be used by the surgeon during the phase of the "intentional" creation of the defect on the cranial case, in order to ensure the best positioning and reduce the geometrical defects due to the manual operations. Specifically, such masks were designed according to the specific anatomical conformation of the sheep; they allowed to minimize the positioning error by the surgeon, making the operations for generating the damage highly repeatable and less affected by positioning errors. Of course, each mask was set according to the hypothetical dimension of the damage, fixed as an elliptical profile, characterized by the size of the major axis and the minor axis respectively equal to 34 mm and 28 mm (the size of the defect, according to what reported in literature, could be considered critical)^30,31^. According to the surgeon’s suggestions, the identification of damage was carried out pointing out 4 reference points that could be easily identified on each specimen. Figure 1 shows the 4 defined points: points 1 and 2 identify an area immediately adjacent to the point of attachment of the nuchal tendons to the skull, whereas locations 3 and 4 (6 mm away from opposite sides with respect to the segment joining the points 1 and 2) identify the sagittal plane. Figure 1 also shows the position of the defect and the mask.

The latter, although custom, also has an elliptical shape with a constant offset of 11 mm with respect to the elliptical edge of the defect. This dimension was chosen as a compromise between minimizing the invasiveness of the surgery and the need to precisely identify the "fitting" of the mask to the skull.

#### Design and manufacturing via SPIF and SPF

**Figure 2:**
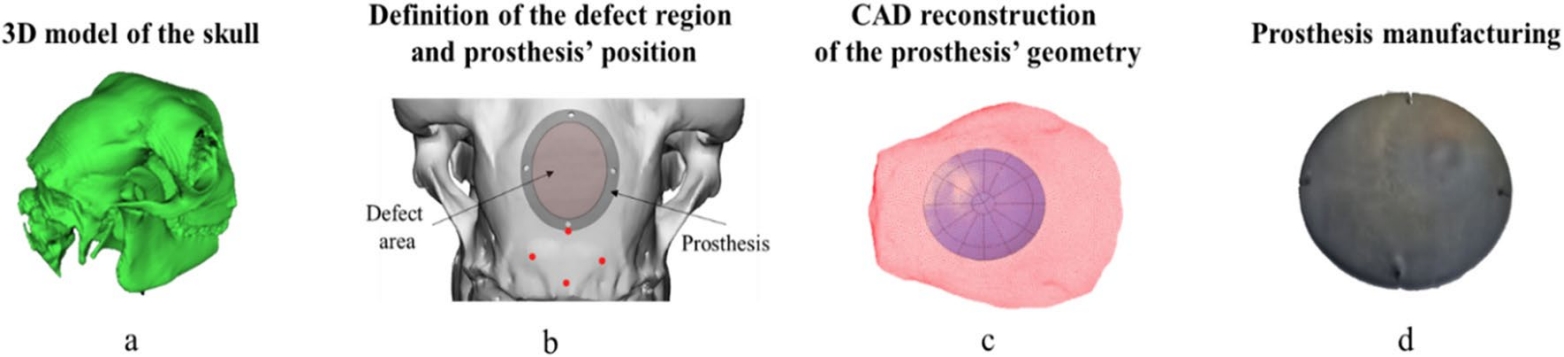
Overview of the design and manufacturing chain for the customized implants.

The prostheses’ manufacturing chain is briefly recalled in Fig. 2 (a more detailed description of the approach is reported in^32^): after the creation of the 3D model from the DICOM files (step “a”), the damage area was defined and located (step “b”). The geometry of prosthesis (step “c”) was designed by offsetting the elliptical shape geometry of the defect of a quantity equal to 4 mm. After the process design based on numerical simulation, the prosthesis was eventually manufactured (step “d”).

The numerical simulations for the process design were run using the commercial code Abaqus/CAE (v. 2017, distributed by Dassault Systemes, https://www.3ds.com/, Vélizy-Villacoublay, France).

**Figure 3:**
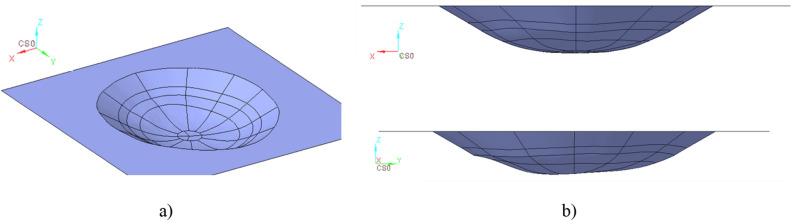
(**a**) 3D view and (**b**) lateral views of the designed geometry for SPIF process.

Regarding the SPIF process, the process design passed through the 3D shape positioning of the flat sheet as well as the tool trajectory to compensate the intrinsic inhomogeneous thickness distribution. An example of one of the CAD models that reproduces the profile for obtaining the prosthesis is reported in Fig. 3.

A CNC milling machine (Mazak Nexus 410), equipped with a heating chamber to increase the working temperature (up to 450 °C), was used to manufacture the implants by SPIF adopting punch diameter of 6 mm whose trajectory was calculated by a CAD/CAM software. The final geometry was isolated from the formed blank and the 4 anchoring holes subsequently drilled. The final part was characterized by a deviation from the designed CAD geometry of around 0.12 (± 0.01 mm) mm^32^.

**Figure 4:**
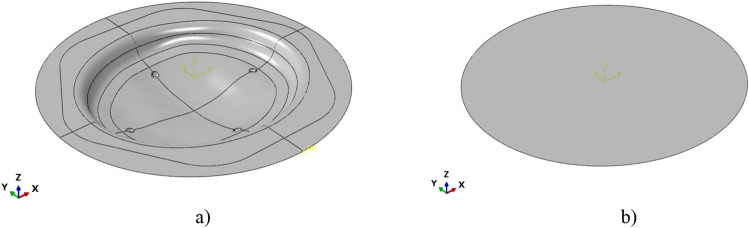
Modelled parts in the FE model of the SPF process: (**a**) the die cavity, (**b**) the Ti blank.

Basically, for the SPF process the CAD geometry of the final implant (the output in Fig. 2, step “c”) was used to determine the geometry of the die cavity to be copied by the blank during the forming operation (see Fig. 4).

**Figure 5:**
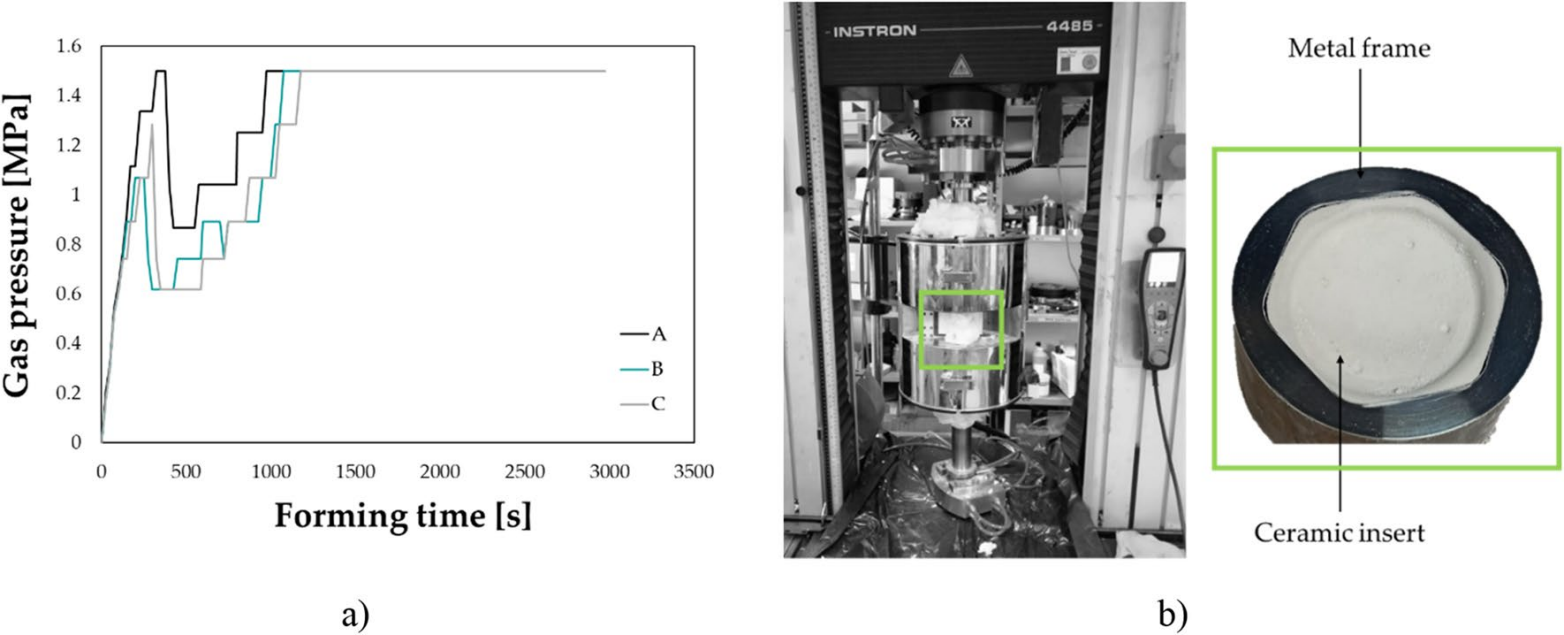
Implant manufacturing by SPF: (**a**) example of the gas pressure profile calculated from the FE simulation of the SPF process, (**b**) experimental equipment.

The numerical process simulation allowed to determine the gas pressure profile able to deform the blank under an optimal strain rate level (the determination of the optimal value came from a former investigation^33^). Figure 5a shows three gas pressure profiles (maximum value 1.5 MPa) used for the manufacturing of three different customized implants.

A universal testing machine (model 4485, produced by Instron, www.instron.com, Norwood, MA, USA) controlled by a dedicated software (testXpert, developed by Zwick/Roell, www.zwickroell.com, Ulm, Germany) and equipped with a specifically designed equipment was used (see Fig. 5b) for the SPF tests. The set-up was heated up to 850 °C by means of an electric split furnace: to reduce the process costs, a cylindrical metal frame containing the ceramic insert with the negative geometry of the prosthesis was adopted. The Ti6Al4V-ELI blank (supplied by RL3 s.r.l., thickness 1 mm, diameter 75 mm) was deformed according to the gas pressure profile determined from the numerical simulation. The final prosthetic geometry (overall geometrical deviation of 0.02 mm with respect to the CAD geometry^32^) was extracted by means of a wire electrical discharge cutting operation. The formed blanks, irrespective of the specific manufacturing route, were characterized by comparable surface roughness in the order of few microns, measured using the Mitutoyo Surftest SV-2000 (Mitutoyo, Kawasaki, Japan).

### In vivo study in sheep model

All methods discussed in the following sections were performed in accordance with the relevant guidelines and regulations on the protection of animals used for scientific purposes (European Directive 2010/63/EU and Italian Law by Decree 26/2014). The in vivo study was conducted at the Department of Emergencies and Organ Transplantation of the University of Bari. The experimental protocol and surgical procedures were firstly approved by the Animal Welfare Body (IRB) of the University of Bari-Aldo Moro and then authorized by The Italian Ministry of Health (authorization number N° 654/2020 PR on March 5, 2019). The study has herein been reported following the ARRIVE guidelines. The sample size of four sheep for each type of prosthesis and experimental time was calculated by using the G*Power software v.3.1.9.6 (Kiel University, Kiel, Germany) and considering a power of 0.80, a type I error α = 0.05, and assuming that one prosthesis could be superior to the other in terms of bone tissue response with an effect size *f* greater than 0.40.

#### Study design

The study evaluated the performance of both SPF and SPIF prosthesis for cranial defect reconstruction in 16 healthy female Bergamasca sheep, (2 years old, 50–60 kg in weight). After selection, animals were housed in the animal facility at the department DETO of the University of Bari with six animals per fence and acclimatise for 15 days prior the surgery. They had free access to water and food, as well as exposure to natural daylight. Feeding was withheld 24 h before surgery.

The sheep were randomized into two study groups to receive either the prostheses SPF (n = 8) or SPIF (n = 8). These were further divided into two subgroups according to experimental time, the first to be euthanized after three months (Subgroup 1; n = 4) and the second after six months (Subgroup 2; n = 4). A sham negative control was not used because the procedures are not consistent with those employed in the present study and would have led to discomfort not justified. As for positive control, the material used in the study is already of proven use in clinical practice, while there is no indication on production processes that can be considered reference standards.

After implantation, the animals were closely monitored: follow-up procedures consisted of daily clinical evaluation of the animals by the veterinary staff of the DETO department. To perform dynamic histomorphometry measurements, sheep received i.m. injections of:

Oxytetracycline fluorochrome (30 mg/kg b.w., Terramicin Long Acting, Zoetis, Italy) administered at 1 month and 4 months after surgery for the Subgroup 1 (3 months) and Subgroup 2 (6 months), respectively; Xylenol Orange (90 mg/kg b.w., Xylenol Orange Tetrasodium Salt, Merck KGaA, Darmstadt, Germany) administered at 2 months (Subgroup 1) or 5 months (Subgroup 2) after surgery; Calcein Blue (30 mg/kg b.w., Calcein Blue, Sigma-Aldrich, St. Louis, MO) administered at 3 months (Subgroup 1 and 2) and Alizarin Red (30 mg/kg b.w. Alizarin-3-methyliminodiacetic acid, Sigma-Aldrich, St. Louis, MO) administered at 6 months after surgery for the Subgroup 2. Animals were sacrificed at the end of the 3- and 6-months follow-up period.

#### Surgical procedure

Surgeries were performed in aseptic conditions. Sheep were placed in lateral decubitus with the head elevated using a positioner and intravenous (i.v.) catheters were placed in the jugular vein after premedicating the sheep with diazepam (0.4 mg/kg i.v., Ziapam ,Domes Pharma SC), methadone (0.3 mg/kg i.v., Domidine Dechra U.K.), ketoprofen (2 mg/kg i.m., Vet Ketofen,Ceva Salute Animale SPA), and amoxicillin/clavulanic acid (10 mg/kg i.m., Sinulox ,Zoetis).

**Figure 6:**
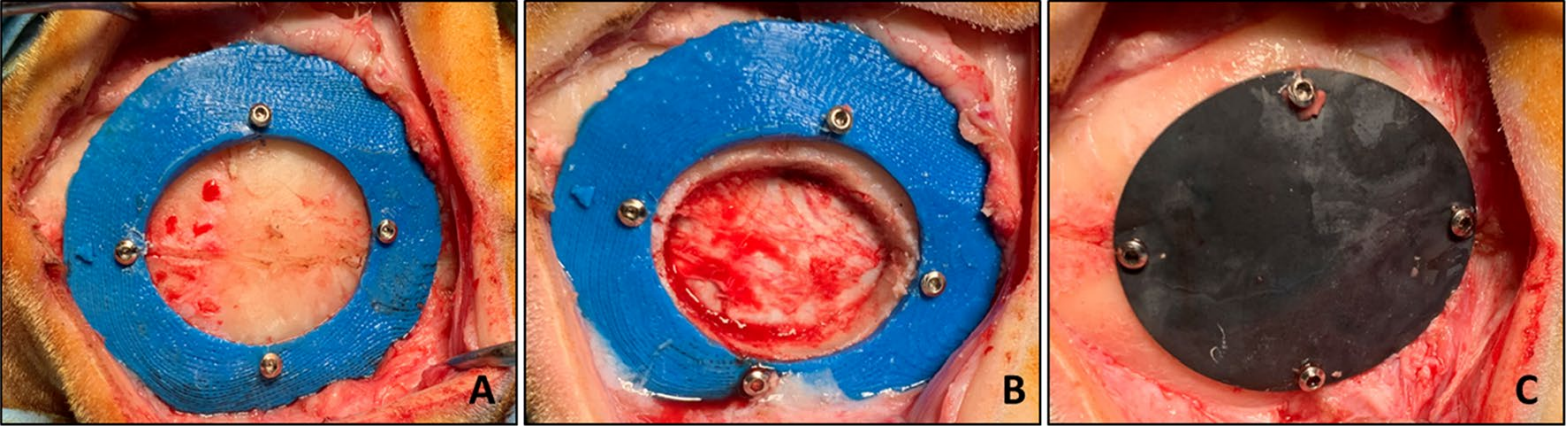
Three distinct moments of different surgeries procedure: (**A**) Guiding mask fixed to the bone with screws; (**B**) Bone gusset removal through Piezosurgery osteotomy; (**C**) Device implantation and fixation with screws.

An incision was then made at the level of the parietal bone, and the custom-made mask was attached to the bone using 2 mm screws (Fig. 6A); the distance between the center of the holes and the inner edge of the guiding mask (corresponding to the geometry of the defect) was equal to 2 mm. Ostectomy through Piezosurgery® (Mectron SpA, Carasco GE, Italy) was then performed (Fig. 6B)^34–36^. The bone gusset and mask were then removed, and the device replaced the bone gusset before having securely fastened it using the same holes (Fig. 6C). Subsequently, tissues were sutured, and the sheep were awakened from the anesthetic state. All the surgery procedures had a duration of approximately 45 min.

#### Anaesthesia and postoperative medication

Anesthesia was induced using an injection of propofol (4–5 mg/kg i.v., Proposure, Boehringer Ingelheim Animal Health Italia SpA, Noventana PD, Italy) and then maintained through an isoflurane and air-oxygen mixture. Additionally, dexmedetomidine (0.5–1 mcg/kg/h, Dexdomitor, Vetoquinol Italia srl) was continuous administered during surgery. Buprenorphine (10mcg/kg; i.m,) and additional amoxicillin/clavulanic acid (10 mg/kg; i.m.) was then given to the animals in the awakening phase. Postoperative medications included Analgesic, anti-inflammatory, and antibiotic treatments (ketoprofen; 2 mg/kg; every 24 h for seven days; buprenorphine; 10mcg/kg; every 12 h for five days; amoxicillin/clavulanic acid; 10 mg/kg; every 12 h for seven days) provided via intramuscular approach the day post-surgery.

#### Euthanasia

Upon reaching the experimental times, sheep were pharmacologically euthanized with an overdose of 20–30 mg/kg propofol, after which the heads were analyzed with CT scans to evaluate the effects of the implant on the bone. A peri-implant bone-crown of about 1–1.5 cm thick was explanted, together with the prosthesis via a craniotomy conducted by Piezosurgery® with an instrument setting of cortical bone cut using an OT 12 tip (micro-bone saw 0.35 mm with a blade having a circular cutting edge).

### Bone sample preparation for microtomography and histology

**Figure 7:**
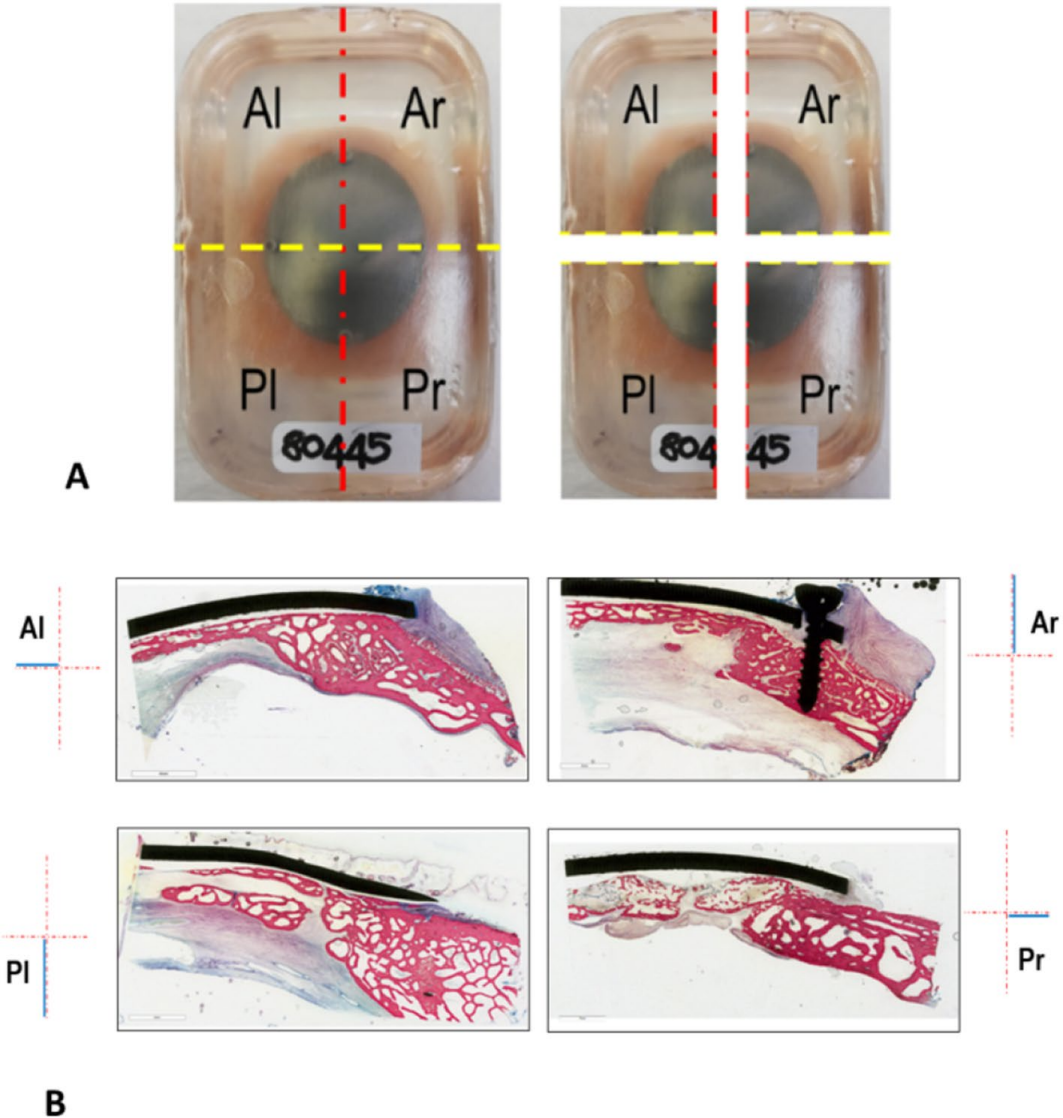
(**A**) Example of specimen embedded in polymethylmethacrylate. The sagittal cutting axis and the transversal cutting axis are highlighted by the red and the yellow dashed lines respectively; (**B**) Representative histological images of Ar and Pl representing the major axis of the prosthetic implant and of Al and Pr corresponding to the minor axis. Stain: Stevenel Blue counterstain with Picrofucsin according to Van Gieson; scale bar: 3 mm.

Architectural characteristics and local histo-pathological response were assessed on resin-embedded specimens by micro-CT and histology, respectively. Bone segments were fixed in 4% buffered paraformaldehyde for seven days, washed under running water for 24 h, and dehydrated in a graded series of alcohol/water solutions for 48 h each (50%, 70% and two passages in 95%), followed by complete dehydration in absolute alcohol (two passages) and finally embedded in polymethylmethacrylate (Merck KGaA, Darmstadt, Germany). The specimens were sectioned along the sagittal axis and the two left and right semicircles obtained were further cut along the transversal axis with the EXAKT 310CP precision cutting system (EXAKT Apparatebau, Nordestedt, Hamburg, Germany), obtaining four quadrants: Ar (Anterior right), Al (Anterior left), Pr (Posterior right) and Pl (Posterior left), as shown in Fig. 7A The left anterior quadrant (Al) of each sample was used for microtomographic evaluations.

Consecutive sections from the medial surfaces of the Ar and Pl quadrants and from the posterior surfaces of the Al and Pr quadrants were abrasively grinded, polished and thinned to a final thickness of 50 ± 10 μm (Saphir 550, ATM GmbH, Mammelzen, Germany). The histological sections of Ar and Pl correspond to the major axis of the prosthetic implant, while those of Al and Pr correspond to the minor axis (Fig. 7B). In particular, the sections related to the major axis concern two different anatomical segments; the parietal bone (Ar) and the occipital bone (Pl) respectively. The sections relating the minor axis concern the right parietal bone (Pr) and the left parietal bone (Al) or, in some cases, the lambdoid suture. Unstained sections were used for dynamic histomorphometry and after having stained with Stevenel Blue and counterstained with Picrofucsin, according to Van Gieson method, were used for static histomorphometric measurements and qualitative histological evaluations.

### Microtomography

**Figure 8:**
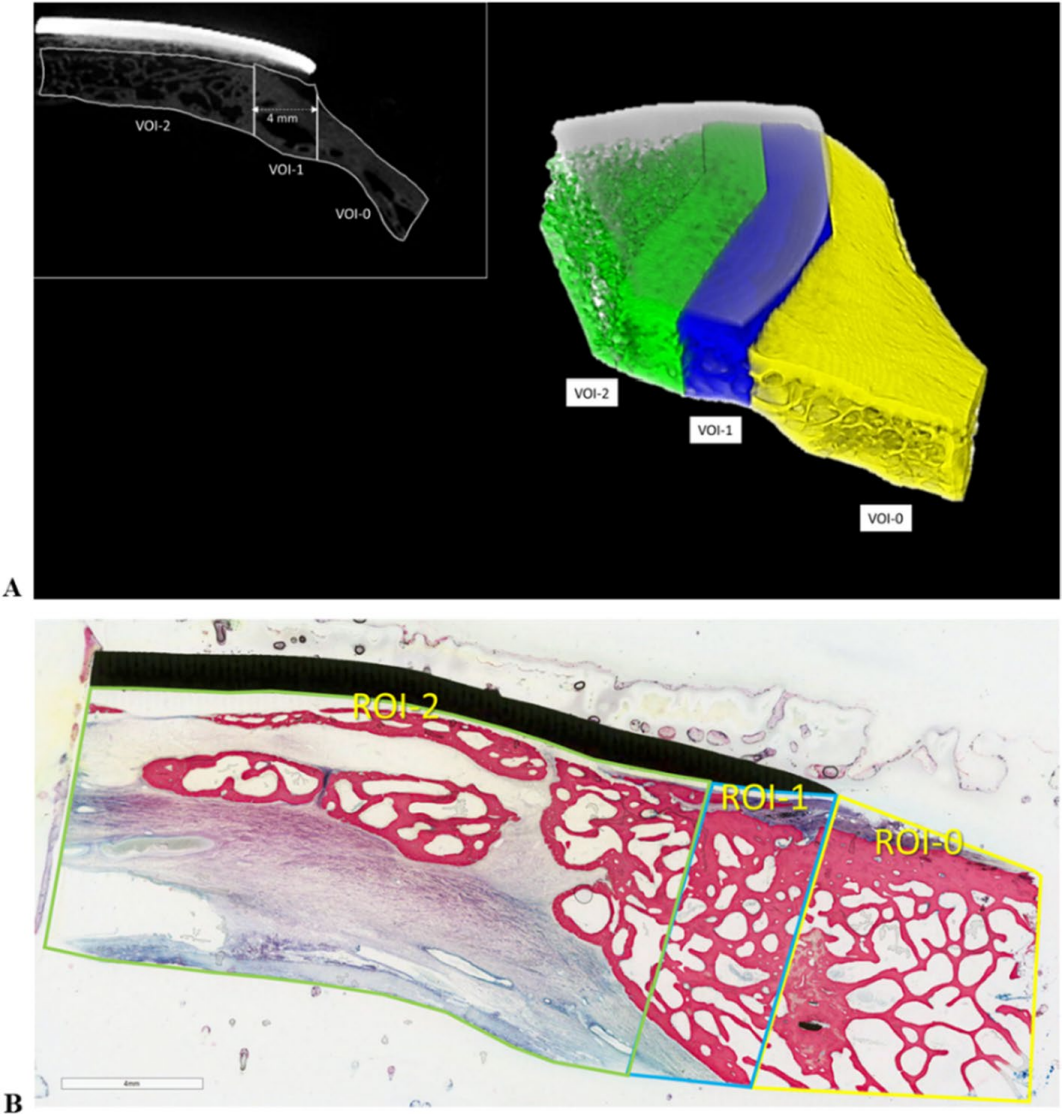
(**A**) left, radial section of the microtomographic reconstruction, highlighting the areas corresponding to the volumes of interest (VOIs): VOI-0 (host bone outside the implant) surrounds the cranial site close to the implant; VOI-1 (host bone inside the implant) is 4 mm wide, i.e. more than half the difference between the implant and defect ellipsoidal axes, starting from the outer edge of the implant and extending towards the centre; VOI-2 (within the defect) starts from the end of VOI-1 and surrounds the regenerated bone. To avoid the influence of metal artefacts, the VOIs were spaced approximately 600 μm from the implant, concentrating on the bone tissue but neglecting any quotes close to the prosthesis, for which reference is made to histology. Again because of the metal artefact, but also because of the difficulty in following the actual host bone margin, the inner margin of VOI-0 and the lateral margins of VOI-1 are drawn geometrically vertical, unlike ROI-0 and ROI-1 in the histology images. On the right, 3D views of a microtomographic reconstruction (obtained with CTVox v.3.3.1 software) with the implant in light grey and the cranial bone in different colours depending on the VOI. (**B**), ROIs definition. ROI-0 (yellow rectangle): cranial bone immediately nearby the implant site; ROI-1 (blue rectangle): portion of the cranial bone below the prosthesis extending from the edge of the prosthesis to the edge of the bone defect; ROI-2 (green rectangle): region corresponding to the bone defect following cranial bone curvature and based on the position of the dura and prosthesis. Scale bar: 4 mm.

All Al samples were scanned in the Skyscan 1172 (Bruker Micro-CT, Belgium) at a nominal resolution (pixel size) of 36 μm employing an aluminium-copper filter 1 mm thick and an applied x-ray tube voltage of 100 kV. Camera pixel binning of 4 × 4 was applied. The scan orbit was 360 degrees with a rotation step of 2 degrees. Reconstruction was carried out with a modified Feldkampii algorithm using the SkyScanTM NRecon software accelerated by GPUiii. Ring artefact reduction and beam hardening correction were applied. Volume of interest selection, segmentation to binary and morphometric analysis were all performed using SkyScan CT-Analyser (“CTAn”) software. Firstly, orienting the reconstructed micro-CT images virtually allowed for the definition of a radial cutting plane that passes between the implant (i.e. defect) centre and the bisector of the inner implant angle with respect to the skull, as viewed from above (Fig. S1). On that plane, the bone tissue was divided into bone volumes of interests (VOIs), numbered in a progressive manner from the cranial site outside the implant (VOI-0) towards the centre of the defect (VOI-1 inside the implant's host bone and VOI-2 at the defect site). Figure 8A depicts the VOIs. To avoid image misreading due to metal artefact around the implant, the analysed VOIs distanced about 600 μm from the implant (Fig. 8A); consequentially, the eventual bone tissue quotes close to the prostheses were ignored in this phase, but considered in the histological one.

For morphometric analysis, the following parameters, defined by the American Society for Bone and Mineral Research (ASBMR), were measured:

- BV/TV (%): percentage of bone volume defined as ratio between bone volume (BV) inside the VOI and total volume (TV) of VOI;
- Tb.Th (mm): trabecular thickness measured starting from the skeletonization of the trabecular structures, identifying their median axis and proceeding to measuring their local thickness by a “sphere-fitting” for all the voxels lying on that axis^37–39^;
- Tb.Sp (mm): trabecular spacing, i.e. the thickness of the spaces inside the VOI;
- Tb.N (1/mm): trabecular number, i.e. the number of crossings for unit of length done by a random linear path through a trabecula inside the VOI.

In addition, a semi-quantitative evaluation of the achieved defect closure was performed. In the central micro-CT radial section, the ratio between the line segment connecting the implant's external margin and the margin of the grown bone, and the line segment parallel to the previous one and connecting the implant's internal and external margins, was defined as a percentage (Fig. S2).

### Static and dynamic histomorphometry

Static histomorphometric measurements were performed by Fiji software, an open-source image processing package based on ImageJ (Schindelin et al. 2012, Schneider et al. 2012). The analysis was carried out on images at a resolution of 1946 × 942 pixels on which 3 regions of interest (ROIs) were identified (Fig. 8B). The histomorphometric parameters were measured as defined by the “American Society of Bone and Mineral Research (ASBMR)”^40^:

- Bone Volume (BV/TV, %): percent of total volume of ROI that is occupied by bone (BV), calculated by applying the following formula:
  $$\frac{B.Ar}{T.Ar}\cdot \frac{4}{\pi }\cdot 100$$
  where *B.Ar* represent the amount of bone measured in the ROI and *T.Ar* the entire area of the ROI considered.
- Trabecular Thickness (Tb.Th, mm) calculated by the following formula:
  $$Tb.Th=\frac{BV/TV}{Tb.N}$$
  where *Tb.N* is the number of trabecula and is calculated as followed:
  $$Tb.N=\frac{1}{2}\cdot \left(\frac{B.Pm}{T.Ar}\cdot \frac{4}{\pi }\cdot 100\right)$$
- Trabecular separation (Tb.Sp, mm) calculated by applying the following formula:
  $$Tb.Sp=\frac{1}{Tb.N}-Tb.Th$$
- Percentage of bone defect repair (%): calculated as the percentage ratio between the distance measured from the margin of the surgical defect at the front of bone growth towards the center of the defect and that measured by the margin of the surgical defect at the center of the defect itself;
- Bone-to-Implant-Contact (BIC, %): the percentage ratio between the length of bone-implant contact and the perimeter of the implant;

Unstained sections were used for dynamic histomorphometry by means of the semiautomatically light/fluorescence microscope (BX51, Olympus Optical Co. Europe GmbH, Germany) connected to the XC50 Olympus digital camera (Olympus Optical). The dynamic histomorphometric analyses were performed by means of Cell*B Soft Imaging System (Olympus Imaging Solutions GmbH). The same software was used for the acquisition of the qualitative histological images. The dynamic histomorphometric paramenters were measured as defined by the “American Society of Bone and Mineral Research (ASBMR)”^40^:

- Mineralizing Surface per Bone Surface (MS/BS, %) calculated by the following formula:
  $$MS/BS=\left(\frac{1}{2}\cdot \frac{sL.Pm}{B.Pm}+\frac{dL.Pm}{B.Pm}\right)\cdot 100$$
  where *sL.Pm/B.Pm* is the length of the single fluorescent band of a fluorochrome expressed in relation to the length of the bone trabecula and *dL.Pm/B.Pm* is the length of the double fluorescent band.
- Mineral Apposition Rate (MAR μm/day): measured as the distance between the midpoints of two consecutive deposited fronts of fluorochrome divided by the time between the labelling period. It represents the average speed at which individual lines of osteoid are mineralized.
- Bone Formation Rate (BFR/BS μm^3^/μm^2^/day): it expresses the measurement of the amount of mineralized bone formed, per unit of bone surface per day. It is calculated adopting the following formula:
  $$BFR/BS=MAR\cdot MS/BS$$

### Statistical analysis

Statistical analysis was conducted using R software v.4.2.1 (R Development Core Team, 2022) and additional R packages: 'lmerTest' v.3.1–3^41,42^. Continuous data were presented as Mean ± standard error of the mean (SE) and at a significant level of *p* < 0.05. Before carrying out the inferential analysis of the data, their normal distribution (Shapiro–Wilk test of normality) and the homogeneity of variance (Levene test) were checked. For the microtomographic measurements, linear models were used with 'type of prosthesis', 'experimental time', 'VOIs analysed' as fixed factors. Therefore, for the repeated static and dynamic histomorphometric measurements obtained from the 4 quadrants of each prosthesis, linear mixed models (LMM) were used by considering ‘type of prosthesis’, ‘experimental time’, ‘ROI analyzed’ and/or ‘time of administration of fluorochromes—fluorochrome time’ data as fixed factors, while ‘quadrants’ was considered as random factor. Pairwise comparisons of the estimated marginal means were performed as a *post-hoc* test to identify any significant differences, adjusting *p*-values according Sidak's correction.

## Results

### Microtomography

**Figure 9:**
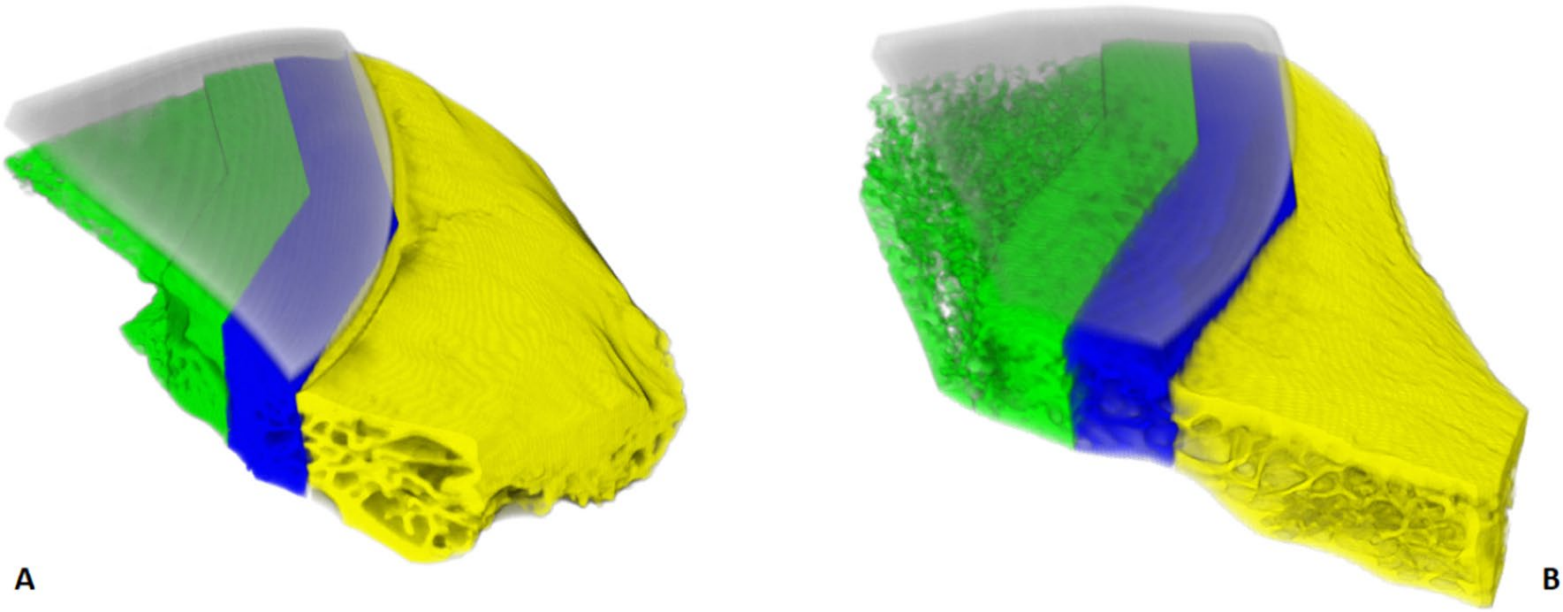
(**A**) 3D rendering of the microtomographic reconstruction of a sample with SPF prosthesis at 6 months. (**B**), 3D rendering of the microtomographic reconstruction of a sample with SPIF prosthesis at 6 months. There are visibly different trabeculae thickness and orientation in VOI-0 (in yellow) for these specific SPF and SPIF examples. That is, the structure of the host bone can have affected the different bone regrowth/remodelling results in respective VOI-1 (in blue) and VOI-2 (in green). However, on average, VOI-0 microtomography morphological parameters did not show statistical differences between SPF and SPIF (see Fig. 10A and Fig. S3).

**Figure 10:**
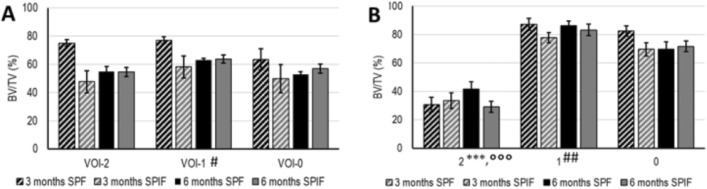
Microtomography (**A**) and histomorphometry (**B**) histograms of BV/TV. (A) reported for experimental times (3 and 6 months), type of prosthesis (SPF and SPIF) and for VOI (VOI-3,-2,-1,-0) or ROI (ROI-2, -1, -0) analyzed. Microtomography LM analysis (Mean ± ES, n = 4). Static histmorphometry LMM analysis (Mean ± ES, n = 16 repeated measures for each type of prosthesis and experimental time): (ROI effect: BV/TV: F = 336.3, *p* < 0.0005. One symbol, *p* < 0.05; 2 symbols, *p* < 0.005; 3 symbols, *p* < 0.0005 : *, ROI-2 *versus* ROI-1; °, ROI-2 *versus* ROI-0; #, VOI-1 (or ROI-1) *versus* VOI-0 (or ROI-0).

Figure 9 illustrates the 3D rendering of a SPF and SPIF prosthesis. In the considered VOIs, morphometry results for percent bone volume (BV/TV) are reported in Fig. 10A while trabecular thickness (Tb.Th), trabecular spacing (Tb.Sp) and trabecular number (Tb.N) are presented in Supplementary Fig. S3.

Statistical analysis revealed a significant interaction between the "type of prosthesis" and "experimental time" factors for BV/TV (F = 13.44, *p* = 0.0007) and for Tb.Th (F = 4.22, *p* = 0.046). Furthermore, a significant effect of the "VOI analysed" factor was observed for BV/TV (F = 3.99, *p* = 0.026), Tb.Sp (F = 3.48, *p* = 0.040) and Tb.N (F = 3.60, *p* = 0.036) and the "type of prosthesis" factor for Tb.Sp (F = 8.13, *p* = 0.007). Pairwise comparisons revealed markedly higher BV/TV values in the SPF group versus SPIF at 3 months (39%, *p* < 0.005) and at 3 months compared to 6 months in SPF (28%, *p* < 0.005) (Fig. 10A). Regardless of experimental time, the BV/TV values were higher in VOI-1 compared to VOI-0 (18%, *p* < 0.05), while Tb.Sp had the opposite trend (− 27%, *p* < 0.05) (Supplementary Fig. S3E).

No significant difference in percentage of defect closure were observed between the SPF and SPIF prostheses between 3 and 6 months. SPF showed a mean closure of 78 ± 12% at 3 months (with 1 of 4 samples showing complete closure) and a mean closure of 95 ± 5% at 6 months (with 3 of 4 samples showing complete closure). Meanwhile, SPIF showed an average closure of 67 ± 16% at 3 months (with 1 of 4 samples showing complete closure) and an average closure of 79 ± 15% at 6 months (with 2 out of 4 showing complete closure).

### Static and dynamic histomorphometry

Regarding static histomorphometric results, Fig. 10B and Fig. S3 report BV/TV (%), Tb.Th (µm), Tb.N (1/mm) and Tb.Sp (µm) measurements. No significant interactions of the selected factors (‘type of prosthesis’, ‘experimental time’, ‘ROI analyzed’) on histomorphometric parameters were found. For BV/TV, significant effects of ‘type of prosthesis’ factor were highlighted (BV/TV: F = 5.12, *p* = 0.025, resulting in significantly higher BV/TV (9%) values for SPF in comparison to SPIF. Significant differences for BV/TV, Tb.Th, Tb.N and Tb.Sp results were observed among ROI analyzed (significant effect values are reported in Fig. 10 and Fig. S3). A significant lower measurement was observed for the BV/TV (%) in ROI-2 respect to ROI-1 (BV/TV: -60%) and ROI-0 (BV/TV: -54%). These values are typical for new bone formation process, characterized by thin trabeculae and wider inter-trabecular spaces due to their spatial distribution in the bone defect. Furthermore, these variations are independent from the type of implanted prosthesis and the experimental time. No significant differences were found between experimental times for all static histomorphometric parameters. As regard BIC (min%—max%—SPF at 3 months: 0–22; SPF at 6 months: 0–39; SPIF at 3 months: 0–3; SPIF at 6 months: 0–29) and percentage of bone defect repair (min%—max%—SPF at 3 months: 13–100; SPF at 6 months: 40–100; SPIF at 3 months: 42–100; SPIF at 6 months: 0–100) results, no significant differences were observed between the types of prosthesis and the experimental times considered.

**Figure 11:**
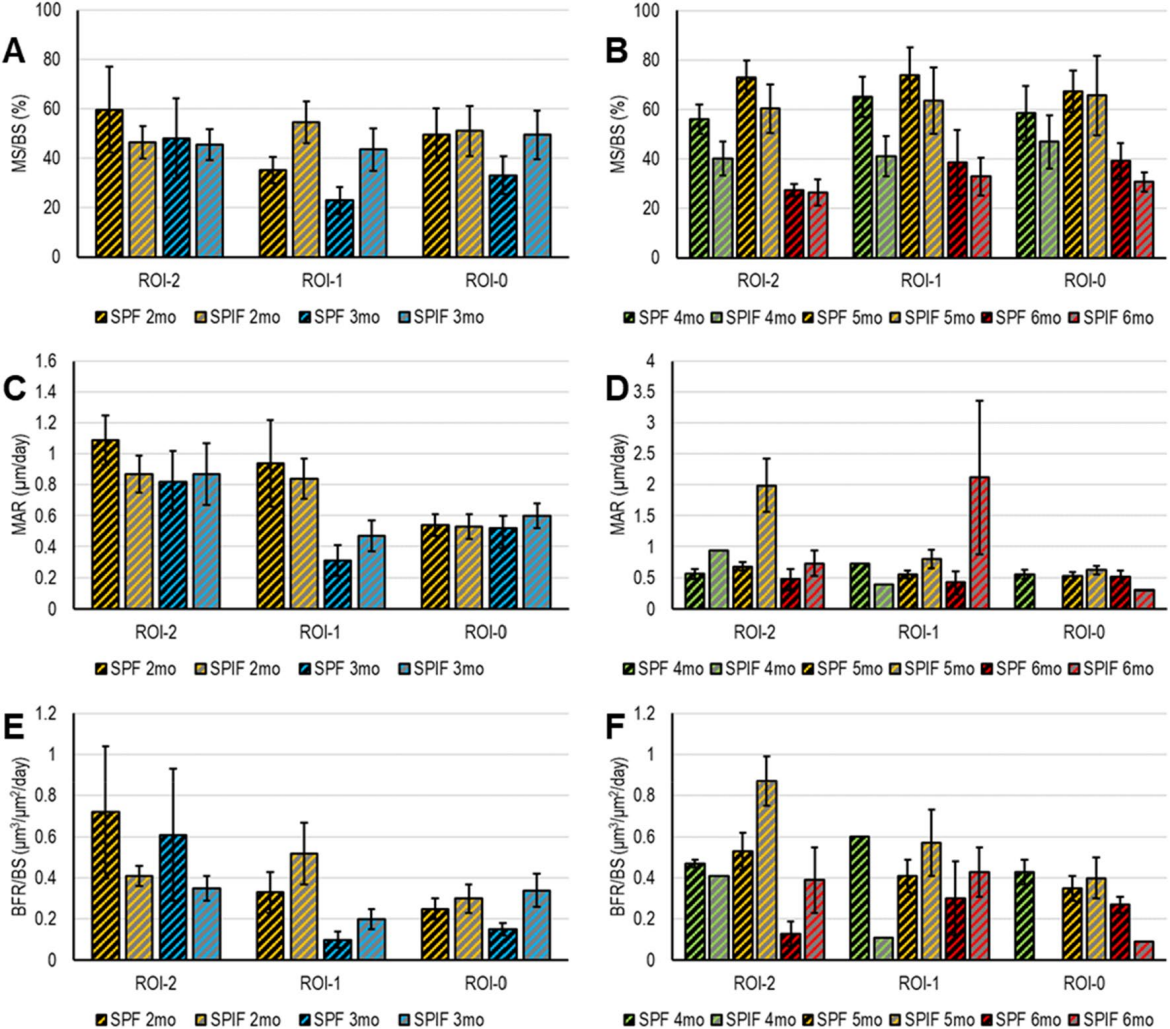
Dynamic histomorphometric results; bar plots of MS/BS (**A**,**B**) MAR (**C**,**D**) and BFR/BS (**E**,**F**) at 3 (**A**,**C**,**E**) and 6 (**B**,**D**,**F**) months, reported for ‘type of prosthesis’ (SPF and SPIF), for ‘ROI analysed’ (ROI-2,-1,-0) and ‘fluorochrome time’ (Mean ± ES, n = 8 repeated measures for each experimental time and type of prosthesis). At 3 months, MS/BS (**A**) ‘type of prosthesis’ effect—F = 5.11, *p* = 0.0026 and ‘fluorochrome time’ effect—F = 4.28, *p* = 0.041; MAR (**C**) ‘fluorochrome time’ effect—F = 7.52, *p* = 0.007 and ‘ROI analysed’ effect—F = 10.25, *p* < 0.0005; BFR/BS (**E**) ‘fluorochrome time’ effect—F = 9.82, *p* = 0.002. At 6 months, MS/BS (**B**) ‘type of prosthesis’ effect—F = 13.27, *p* < 0.0005 and ‘fluorochrome time’ effect—F = 35.17, *p* < 0.0005; MAR (**D**) ‘type of prosthesis’ effect—F = 16.34, *p* < 0.0005; BFR/BS (**F**) ‘type of prosthesis’ effect—F = 11.00, *p* = 0.0014 and ‘fluorochrome time’ effect—F = 6.39, *p* = 0.0027.

Dynamic histomorphometric analysis was carried out on unstained sections observed at ultraviolet light **(**UV**)**; Supplementary Fig. S4 displays a representative image of each type of prosthesis for each experimental time. Due to differences in the experimental time in which the fluorochromes were administered to the animals, statistical analysis of the dynamic histomorphometry data was conducted separately for the data gathered at 3 and 6 months (Fig. 11). The statistical analysis revealed that there were no significant interactions between the factors that were considered, namely 'type of prosthesis', 'ROIs analyzed', and 'fluorochrome time', regarding dynamic histomorphometric parameters. However, the effects of these factors are shown in the legend of Fig. 11. After 3 months, the growth of the bone mineralization surface area (MS/BS) was found to have slowed down (-18%, *p* = 0.041) when compared to 2 months, and the MS/BS results of the SPIF prosthesis indicated a higher value (26%, *p* = 0.026) than those of the SPF. At 5 months, there was an increase in MS/BS of approximately 33% (*p* < 0.0005) compared to 4 weeks, with a subsequent decrease of 54% (*p* < 0.0005) at 6 months; between 3 and 6 months, the SPF prosthesis had higher MS/BS values (30%, *p* < 0.0005) compared to the SPIF. Furthermore, there was a 26% reduction in MAR values at 3 months compared to 2 months (*p* = 0.007), with higher mineral apposition activity in ROI-2 compared to ROI-1 (54%, *p* < 0.005) and ROI-0 (53%, *p* < 0.005). The sole noteworthy difference in MAR at 6 months was the higher rate with the SPF prosthesis compared to SPIF (30%, *p* < 0.0005). Finally, a 33% (*p* = 0.002) and 28% (*p* = 0.003) reduction in BFR/BS was observed at 3 months compared to 2 months and 6 months compared to 5 months, respectively. Additionally, at 6 months, the BFR/BS values of the SPIF prostheses were 57% (*p* = 0.002) higher than those of the SPF prostheses.

### Histology

**Figure 12:**
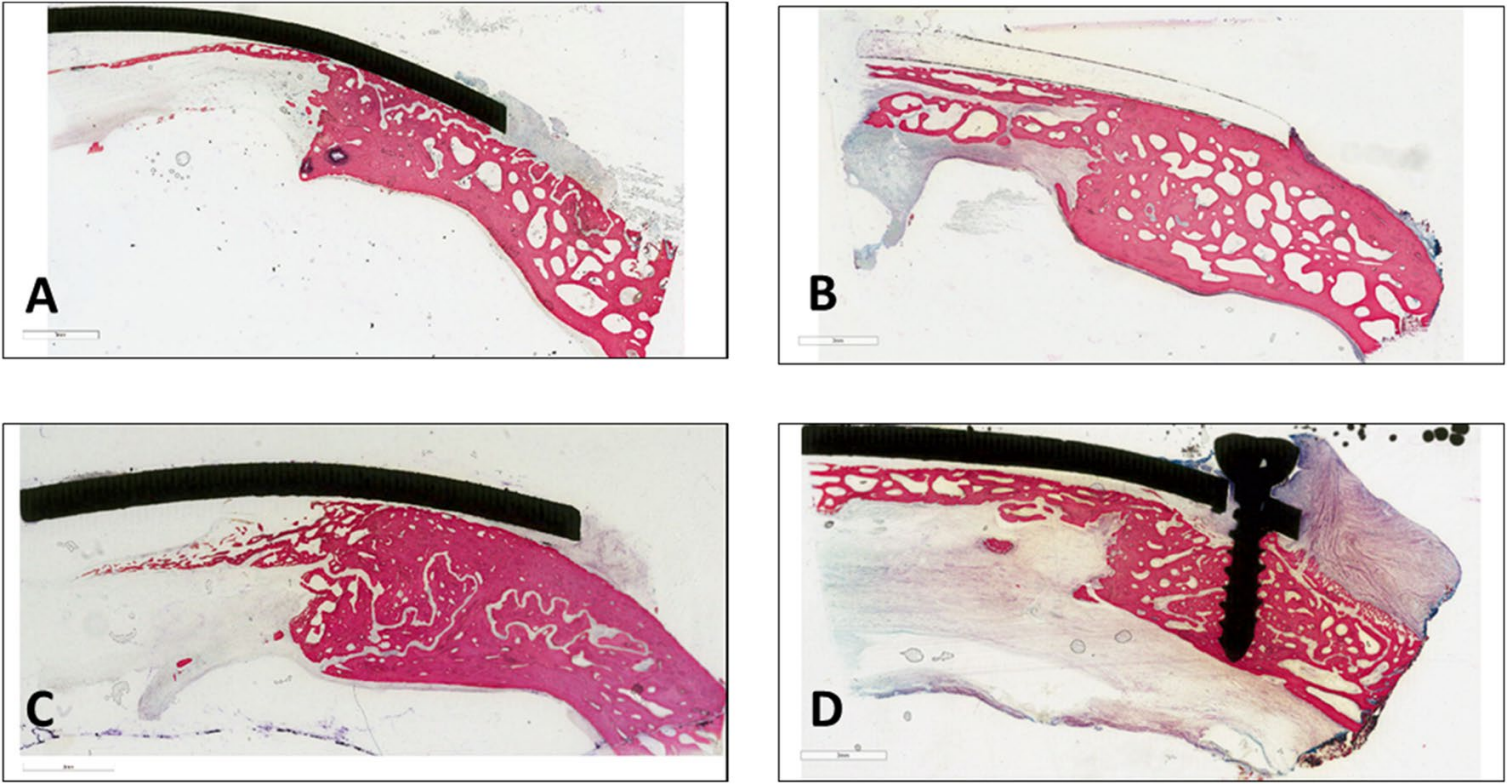
Histological images of SPF prosthesis at 3 months (**A**) and 6 months (**B**) and of SPIF prosthesis at 3 months (**C**) and 6 months (**D**). Staining: Stevenel Blue counterstain with Picrofucsin according to Van Gieson; scale bar: 3 mm.

Figure 12 shows representative histological images of the SPF samples at 3 (Fig. 12A) and 6 months (Fig. 12B) and of the SPIF samples at the same experimental times (3 months: Fig. 12C; 6 months: Fig. 12D).

**Figure 13:**
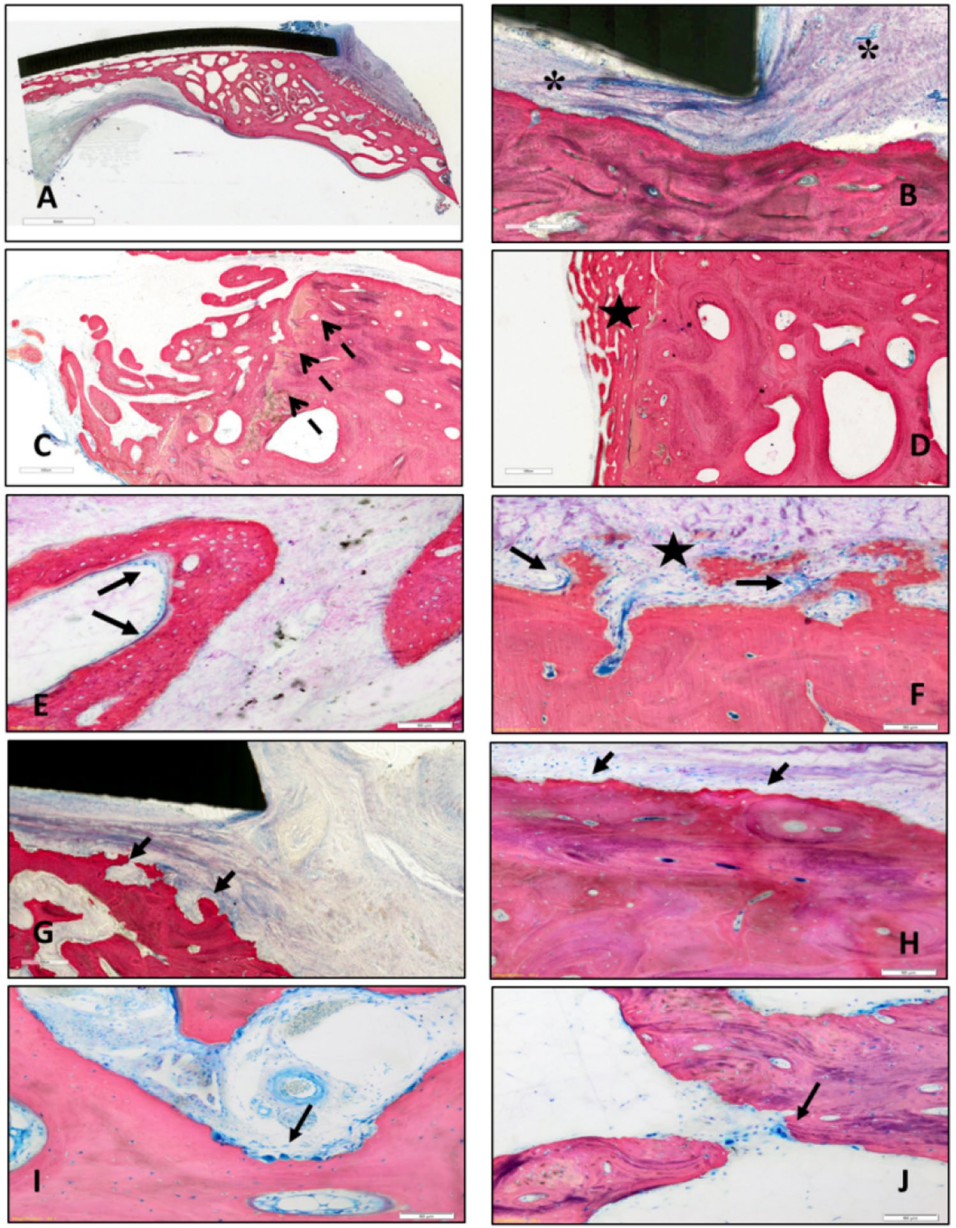
Representative histological highlights; for each image the top side is that facing the prosthesis: (**A**,**B**; ROI-0), connective tissue separating the inner part of the prosthesis from the bone tissue below (*); (**C**,**D**; ROI-1), bone growth front characterized by more disorganized tissue architecture with thinner trabeculae (black star) and necrotic tissue localized in correspondence to the margin of surgical defect (dotted arrows); (**E**,**F**; ROI-2), osteoblastic activity (black arrows) and bone remodelling (black star); (**G**,**H**; ROI-0), remodelling phenomena of the external cortical bone; (**I**, ROI-0; **J**, ROI-1), erosions zones characterized by areas of osteoclasts (black arrows). Staining: Stevenel Blue counterstain with Picrofucsin according to Van Gieson; Scale bar: (**A**), 4 mm; (**B**), 300 μm (**C**,**D**), 500 μm; (**G**), 200 μm; (**E**,**F**,**H**–**J**), 50 μm.

The prostheses were well recognizable at both experimental times and in some cases one of the screws for fixing them was visible (Fig. 12D). In few sections, only the impression of the prosthesis was visible, resulting from the grinding and polishing processes of the histological specimens (Fig. 12B). Moving from the outer portion of the defect (where the prosthesis rests on the cortical bone) to the centre of it, different histological patterns were seen in correspondence of the interface between the host bone and the prosthesis. In some sections bone growth was observed in proximity mainly with the prosthetic portion corresponding to the ROI-1 region, in correspondence with the support point. No direct apposition of newly formed bone was observed in contact with the prosthesis in most of the sections: connective tissue separating the inner part of the prosthesis from the bone tissue below was present and, in some cases, it formed a fibrous capsule, which surrounded the outer edge of the prosthesis (Fig. 13A,B). A bone growth front that extends from the margin of the surgical defect (ROI-1) toward the centre of the defect, partially involving portions of ROI-2, was clearly visible. This bone growth front is evidenced by the picrofucsin staining, which highlights the newly formed bone with a more intense colour than the pre-existing one, by the more disorganized tissue architecture with thinner trabeculae and by the presence of necrotic tissue exactly localized in correspondence to the margin of the surgical defect created (Fig. 13C,D). Islets of new bone were also spotted in certain samples that lacked contact with the native bone, specifically in the ROI-2 on the dura side (Fig. S5).

As evidenced by Stevenel’s Blue staining, the new bone formed in correspondence of the defect was characterized by an intense osteoblastic activity resulting in new bone matrix deposition and different mineralization grade due to bone remodeling (Fig. 13E,F). Furthermore, remodelling phenomena of the external cortical bone, in the contact zone between the prosthesis and the peri-implant host bone (Fig. 13G,H) were observed. These phenomena were characterized by the concomitant presence of areas of cuboidal osteoblast cells that lay bone matrix and areas of osteoclasts in correspondence of erosion zones (Fig. 13I,J).

## Discussion

Two highly customized prostheses, manufactured using SPF and SPIF techniques, were evaluated microtomographically and histologically for their effect on bone tissue in an in vivo craniectomy sheep model. The study examined inflammatory reactions, new bone deposition, and repair of bone defects. The manufacturing process used to create titanium prostheses was previously demonstrated in other studies^1,23^. Briefly, the conversion of the DICOM images to CAD models improves the optimal definition of the implant's geometry. SPF implants, where found to be more geometrically accurate (with an average deviation from CAD geometry of only 0.02 mm) when compared to those manufactured by SPIF. However, they required a longer manufacturing time due to the need for pre-heating of the tools and a longer forming step. Furthermore, the creation of the guiding mask to ensure accurate implant positioning during surgery, minimized the risk of micro-motion and mismatch issues with the surrounding peri-implant bone. These adopted strategies were able to guarantee the most suitable prosthesis fit to sheep skull anatomy^1^.

No significant inflammatory response or side effects were observed in the implant site. Additionally, the healing of soft tissue was normal and aligned with expected scarring times. The healing processes of craniomaxillofacial bone defects have been extensively documented in the literature across various animal species^43^. Ratsand rabbits are the most commonly used preclinical animal models, but sheep have bone dimensions similar to those in humans, along with comparable turnover and bone modeling rates^44^. When the defect size exceeds 22 mm in diameter, it is considered a critical size defect in sheep^45^. Hubbe et al. replicated this defect in an ovine model’s skull and covered it with a 3D-printed porous titanium-based scaffold. The scaffold was either coated with hydroxyapatite (HA) or left uncoated, and filled with or without bone cement. Histological analyses showed that bone ingrowth was significantly higher in the HA-coated group, starting from the lateral side of the defect, and extending up to the scaffold center^30^.

According to the current microtomographic and histomorphometric results, bone formation was found to start from the native bone, although full-thickness closure of the cranial theca was not observed in any of the sixteen animals. Omar et al., carried out the regeneration of large ovine cranial defects using 3D-printed cranial implants consisting of a titanium-reinforced bioceramic (BioCer) in comparison to titanium control materials^46^. After 12 months, titanium implants exhibited less bone restoration and BIC values (center, 3%; periphery, 19%) compared to BioCer implants which promoted a higher degree of bone formation, remodeling and osseointegration. However, they observed the same bone formation mode reported in this study that primarily occurred along the existing borders of the bone, with a lesser extent in the defect center. As well as, Gallinetti et al.^47^ studied the repair of cranial defects in sheep using a titanium mesh either uncoated or coated with calcium phosphate (CaP). Significantly higher bone neoformation (p < 0.001) with moderate osteoconduction (p < 0.001) and a marked osteointegration (p < 0.001) were observed in the CaP-Ti cover versus the Ti-mesh. They also observed that bone formation started from the native bone and complete closure did not occur in any animal. Additionally, they confirmed that bone growth mainly occurred on the dura side rather than in close proximity to the implants, which were observed on the skin side. This aligns with our histological discovery of new bone growth spots near the dura side but not in direct contact with the native bone. This is likely due to the presence of skeletal stem cells (SSCs) in the periosteal layer of the dura mater, which play a key role in bone regeneration^48,49^. The osteopromoting effects of the dura mater are also reported in the study of Piitulainen et al., where a large calvarial bone defect of a child was reconstructed with a bioactive and biostable nonmetallic implant. New bone was only found on the inner surface of the implant, suggesting the importance of chemical and biological engagement of the dura mater in the regeneration of the bone defect^50^. Despite the SPF and SPIF implants in this study mimicking the shape of the cranial vault and possessing adequate mechanical properties, they differ from the aforementioned studies in that they do not completely fill the entire defect thickness, lack a porous structure to promote vascularization and bone ingrowth and are not reinforced with osteoinductive and absorbable material such as CaP. Furthermore, they have a smooth surface that does not promote cell adhesion.

The cranial defects repaired without direct contact with the prosthetic devices. This is shown by Figs. 8 and 12, where a narrow space is easily detectable between the re-grown bone and the inner surface of the Ti implant. Qualitative analysis the histological images concluded that the newly generated bone followed the curvature of the brain rather than the shape of the Ti implant. In particular, no osteoconductivity properties were observed near the implants, which were still separated by a thin layer of fibrous tissue. Connective tissue growth underneath the implants could be caused by various factors, including inadequate peripheral healing (i.e. anchoring point) between the material and the bone, resulting in connective tissue ingrowth through the external margins. These results suggest that SPF and SPIF implants do not directly participate and stimulate bone regeneration in the cranial defect. Instead, they provide necessary protection and rigidity, acting as a barrier membrane in guided bone regeneration (GBR), thus sustaining the space of the regenerating bone area beneath it^51–53^. Due to its excellent mechanical properties, titanium frameworks are commonly used in the dental GBR technique, mostly embedded inside polytetrafluoroethylene (PTFE) membranes in order to provide additional stability and adaptation to the bone defect^54^. The decision to employ titanium in GBR drew inspiration from the positive results achieved when utilizing a titanium mesh to reconstruct maxillofacial defects^55–59^. Finally, bone resorption observed on the external surface of the cranial theca, specifically where the prosthesis is implanted on native bone, it may occur due to physiological remodelling activity of the cortical bone in response to mechanical stresses, such as stress-shielding, experienced by the fixation screws.

A possible limitation of the current study could be the lack of control implants, but to our knowledge the available options for cranioplasty, apart from autologous bone grafts, are typically titanium-based (like our devices) or made from acrylic materials^60^. The analysis of the literature, with particular reference to clinical studies, supports that there is no standard production process for cranial prosthesis, due to the use of diverse surgical approaches, materials, and manufacturing methods. Studies are predominantly oriented towards the use of methods that allow the creation of customized devices, based on preoperative CT imaging, overcoming the use of prefabricated prostheses to be shaped in a surgical procedure. Even comparing various titanium implant production methods does not yield a clear universal approach for both customized and non-customized prostheses. This entails an ethical and scientific difficulty in selecting a reliable and recognizable control. Therefore, the design of the present pilot study, relying on the use of device produced using a material with proven biocompatibility and orthopaedic effectiveness such as titanium, did not foresee a further group with prefabricated prostheses^61–64^. Furthermore, according to the principles of the 3 R's (Refinement, Reduction, Replacement) and in our opinion, the inclusion of a control material group would have had a further impact on the total number of animals used rather than the results of the study. Similarly, not comparing the results with a sham defect could be considered a limitation. Our decision to omit such control defects, since the sham technique involves craniotomy, removal of the bone segment and its immediate repositioning and fixation to the skull. In addition, the method of attachment of the autogenous graft to the skull would have been different from that used in the prostheses tested, which would have reduced the ability to withstand external mechanical stresses of a pressor type. Such stresses could be caused by social communication behaviour or scratching and itching of the sheep, with the risk of damage to brain structures. Further investigations could be implemented in future studies to provide a more detailed and in-depth characterization of the quality and state of the regenerated bone tissue. Techniques such as Raman spectroscopy or FTIR could be used for this purpose.

## Conclusions

In this study, a novel approach combining mechanical and surgical techniques was proposed. The adoption of two innovative and flexible sheet metal forming processes – the SPF and the SPIF allowed to achieve the geometrical complexity necessary to ensure a high level of correspondence between the formed components and the bone’s region surrounding the defect.

The conducted in vivo test highlights the absence of any adverse effects or inflammatory reactions, indicating strong mechanical fixation of the prosthesis to the adjacent bones, serving as evidence for the high precision achieved through this methodology. The implants under study offer indirect support for bone regeneration by providing protection and rigidity. Bone filling involves from 30 to 40% of the defect and primarily follows the natural curve of the cranium in relation to the covering of the brain.

### Supplementary Information

Supplementary Information.

## Data Availability

The datasets generated during and/or analysed during the current study are available in the Figshare repository, https://figshare.com/s/af18eba6370c7cff3758.

## References

[1] Palumbo G (2022). A structured approach for the design and manufacturing of titanium cranial prostheses via sheet metal forming. Metals.

[2] Morselli C (2019). Comparison between the different types of heterologous materials used in cranioplasty: a systematic review of the literature. J. Neurosurg. Sci..

[3] Beri AJ, Pisulkar SG, Bansod AV, Dahihandekar C (2022). Paradigm shift in materials for skull reconstruction facilitated by science and technological integration. Cureus.

[4] Ambrogio G (2018). Experimental investigation of the mechanical performances of titanium cranial prostheses manufactured by super plastic forming and single-point incremental forming. Int. J. Adv. Manuf. Technol..

[5] Zanotti B (2016). Cranioplasty. J. Craniofac. Surg..

[6] Mousa MM, Eissa SAF, Raslan MS, Abu ElNaga BF, Balaha AM (2021). Evaluation of three different methods of cranioplasty; a comparative prospective randomized study. Pan Arab J. Neurosurg..

[7] Iaccarino C (2015). Preliminary results of a prospective study on methods of cranial reconstruction. J. Oral Maxillofac. Surg..

[8] Fricia M (2015). Osteointegration in custom-made porous hydroxyapatite cranial implants: from reconstructive surgery to regenerative medicine. World Neurosurg..

[9] Brogini S (2021). Osseointegration of additive manufacturing Ti-6Al-4V and Co-Cr-Mo alloys, with and without surface functionalization with hydroxyapatite and type I collagen. J. Mech. Behav. Biomed. Mater..

[10] Zhu S (2021). Complications following titanium cranioplasty compared with nontitanium implants cranioplasty: A systematic review and meta-analysis. J. Clin. Neurosci..

[11] Tanveer W, Ridwan-Pramana A, Molinero-Mourelle P, Koolstra JH, Forouzanfar T (2021). Systematic review of clinical applications of cad/cam technology for craniofacial implants placement and manufacturing of nasal prostheses. Int. J. Environ. Res. Public Health.

[12] Castelan J (2014). Manufacture of custom-made cranial implants from DICOM?? Images using 3D printing, CAD/CAM technology and incremental sheet forming. Rev. Bras. Eng. Biomed..

[13] Ambrogio G, Conte R, De Napoli L, Fragomeni G, Gagliardi F (2015). Forming approaches comparison for high customised skull manufacturing. Key Eng. Mater..

[14] Cheng Z (2020). Incremental sheet forming towards biomedical implants: A review. J. Mater. Res. Technol..

[15] Zhang J (2023). Additively manufactured polyether ether ketone (PEEK) skull implant as an alternative to titanium mesh in cranioplasty. Int. J. Bioprint.

[16] Harding A, Pramanik A, Basak AK, Prakash C, Shankar S (2023). Application of additive manufacturing in the biomedical field: A review. Ann. 3D Print. Med..

[17] Chua K, Khan I, Malhotra R, Zhu D (2021). Additive manufacturing and 3D printing of metallic biomaterials. Eng. Regener..

[18] Ngo TD, Kashani A, Imbalzano G, Nguyen KTQ, Hui D (2018). Additive manufacturing (3D printing): A review of materials, methods, applications and challenges. Composites B.

[19] Singh S, Ramakrishna S, Singh R (2017). Material issues in additive manufacturing: A review. J. Manuf. Process..

[20] Allavikutty R, Gupta P, Santra TS, Rengaswamy J (2021). Additive manufacturing of Mg alloys for biomedical applications: Current status and challenges. Curr. Opin. Biomed. Eng..

[21] Kumar R, Kumar M, Chohan JS (2021). The role of additive manufacturing for biomedical applications: A critical review. J. Manuf. Process..

[22] Karunakaran R, Ortgies S, Tamayol A, Bobaru F, Sealy MP (2020). Additive manufacturing of magnesium alloys. Bioact. Mater..

[23] Piccininni A (2016). Biomedical titanium alloy prostheses manufacturing by means of superplastic and incremental forming processes. MATEC Web Conf..

[24] Sorgente D, Palumbo G, Piccininni A, Guglielmi P, Aksenov S (2017). Investigation on the thickness distribution of highly customized titanium biomedical implants manufactured by superplastic forming. CIRP J. Manuf. Sci. Technol..

[25] Palumbo G (2018). Effects of superplastic forming on modification of surface properties of Ti alloys for biomedical applications. J. Manuf. Sci. Eng..

[26] Bernhart G, Lours P, Cutard T, Velay V, Nazaret F, Giuliano G (2011). Processes and equipment for superplastic forming of metals. Superplastic Forming of Advanced Metallic Materials Methods and Applications.

[27] Cooper DR, Rossie KE, Gutowski TG (2017). The energy requirements and environmental impacts of sheet metal forming: An analysis of five forming processes. J. Mater. Process. Technol..

[28] Oleksik V (2021). Single-point incremental forming of titanium and titanium alloy sheets. Materials.

[29] Werner Dankwort C, Weidlich R, Guenther B, Blaurock JE (2004). Engineers’ CAx education—it’s not only CAD. Comput.-Aided Des..

[30] Hubbe U (2022). A fully ingrowing implant for cranial reconstruction: Results in critical size defects in sheep using 3D-printed titanium scaffold. Biomater. Adv..

[31] Viljanen VV, Gao TJ, Lindholm TC, Lindholm TS, Kommonen B (1996). Xenogeneic moose (Alces alces) bone morphogenetic protein (mBMP)-induced repair of critical-size skull defects in sheep. Int. J. Oral Maxillofac. Surg..

[32] Palumbo G (2022). A structured approach for the design and manufacturing of titanium cranial prostheses via sheet metal forming. Metals (Basel).

[33] Sorgente D, Palumbo G, Piccininni A, Guglielmi P, Aksenov SA (2018). Investigation on the thickness distribution of highly customized titanium biomedical implants manufactured by superplastic forming. CIRP J. Manuf. Sci. Technol..

[34] Crovace AM (2020). Minimal invasive piezoelectric osteotomy in neurosurgery: Technic, applications, and clinical outcomes of a retrospective case series. Vet. Sci..

[35] Grauvogel J (2018). Piezosurgery: A safe technique to perform lateral suboccipital craniotomy?. Oper. Neurosurg. (Hagerstown).

[36] Stelzle F (2014). The effect of load on heat production, thermal effects and expenditure of time during implant site preparation - an experimental ex vivo comparison between piezosurgery and conventional drilling. Clin. Oral Implants Res..

[37] Hildebrand T, Rüegsegger P (1997). A new method for the model-independent assessment of thickness in three-dimensional images. J. Microsc..

[38] Ulrich D, van Rietbergen B, Laib A, Rüegsegger P (1999). The ability of three-dimensional structural indices to reflect mechanical aspects of trabecular bone. Bone.

[39] Remy E, Thiel E (2002). Medial axis for chamfer distances: computing look-up tables and neighbourhoods in 2D or 3D. Pattern Recognit. Lett..

[40] Parfitt AM (1987). Bone histomorphometry: Standardization of nomenclature, symbols, and units: Report of the asbmr histomorphometry nomenclature committee. J. Bone Miner. Res..

[41] Kuznetsova A, Brockhoff PB, Christensen RHB (2017). lmerTest Package: Tests in linear mixed effects models. J. Stat. Softw..

[42] Russell A (2023). Emmeans: Estimated marginal means, aka least-squares means. Compr. R. Arch. Netw..

[43] Hatt LP, Thompson K, Helms JA, Stoddart MJ, Armiento AR (2022). Clinically relevant preclinical animal models for testing novel cranio-maxillofacial bone 3D-printed biomaterials. Clin. Transl. Med..

[44] Zhang Z, Gan Y, Guo Y, Lu X, Li X (2021). Animal models of vertical bone augmentation (Review). Exp. Ther. Med..

[45] Szpalski C, Barr J, Wetterau M, Saadeh PB, Warren SM (2010). Cranial bone defects: Current and future strategies. Neurosurg. Focus.

[46] Omar O (2020). In situ bone regeneration of large cranial defects using synthetic ceramic implants with a tailored composition and design. Proc. Natl. Acad. Sci. U S A.

[47] Gallinetti S (2021). Titanium reinforced calcium phosphate improves bone formation and osteointegration in ovine calvaria defects: A comparative 52 weeks study. Biomed. Mater..

[48] Hobar PC, Masson JA, Wilson R, Zerwekh J (1996). The importance of the dura in craniofacial surgery. Plast. Reconstr. Surg..

[49] Duchamp De Lageneste O (2018). Periosteum contains skeletal stem cells with high bone regenerative potential controlled by Periostin. Nat. Commun..

[50] Piitulainen JM, Posti JP, Vallittu PK, Aitasalo KM, Serlo W (2019). A large calvarial bone defect in a child: Osseointegration of an implant. World Neurosurg..

[51] Langdahl B, Ferrari S, Dempster DW (2016). Bone modeling and remodeling: Potential as therapeutic targets for the treatment of osteoporosis. Ther. Adv. Musculoskelet. Dis..

[52] Zheng J (2022). Biphasic mineralized collagen-based composite scaffold for cranial bone regeneration in developing sheep. Regen. Biomater..

[53] Rakhmatia YD, Ayukawa Y, Furuhashi A, Koyano K (2014). Microcomputed tomographic and histomorphometric analyses of novel titanium mesh membranes for guided bone regeneration: a study in rat calvarial defects. Int. J. Oral Maxillofac. Implants.

[54] Elgali I, Omar O, Dahlin C, Thomsen P (2017). Guided bone regeneration: Materials and biological mechanisms revisited. Eur. J. Oral Sci..

[55] Boyne PJ (1969). Restoration of osseous defects in maxillofacial casualties. J. Am. Dent. Assoc..

[56] Omar O, Elgali I, Dahlin C, Thomsen P (2019). Barrier membranes: More than the barrier effect?. J. Clin. Periodontol..

[57] Proussaefs P, Lozada J (2006). Use of titanium mesh for staged localized alveolar ridge augmentation: clinical and histologic-histomorphometric evaluation. J. Oral Implantol..

[58] Roccuzzo M, Ramieri G, Bunino M, Berrone S (2007). Autogenous bone graft alone or associated with titanium mesh for vertical alveolar ridge augmentation: a controlled clinical trial. Clin. Oral Implants Res..

[59] Funato A, Ishikawa T, Kitajima H, Yamada M, Moroi H (2013). A novel combined surgical approach to vertical alveolar ridge augmentation with titanium mesh, resorbable membrane, and rhPDGF-BB: A retrospective consecutive case series. Int. J. Periodont. Restor. Dent..

[60] Capitelli-McMahon H, Kahlar N, Rahman S (2023). Titanium versus autologous bone-based cranioplasty: A systematic review and meta-analysis. Cureus.

[61] Policicchio D (2020). Comparison of two different titanium cranioplasty methods: Custom-made titanium prostheses versus precurved titanium mesh. Surg. Neurol. Int..

[62] Mukherjee S, Thakur B, Haq I, Hettige S, Martin AJ (2014). Complications of titanium cranioplasty: A retrospective analysis of 174 patients. Acta Neurochir..

[63] Meyer H, Khalid SI, Dorafshar AH, Byrne RW (2021). The Materials utilized in cranial reconstruction: Past, current, and future. Plast. Surg. (Oakv).

[64] Peel S, Eggbeer D, Burton H, Hanson H, Evans PL (2018). Additively manufactured versus conventionally pressed cranioplasty implants: An accuracy comparison. Proc. Inst. Mech. Eng. H.

