# Supplementary material for: In vivo validation of highly customized cranial Ti-6AL-4V ELI prostheses fabricated through incremental forming and superplastic forming: an ovine model study
Source: Sci Rep. 2024 Apr 4;14:7959. doi: 10.1038/s41598-024-57629-3 (PMC10995190; doi:10.1038/s41598-024-57629-3)
Supplement: Supplementary file 1 — Supplementary Information. [file 41598_2024_57629_MOESM1_ESM.docx]

SUPPLEMENTARY FILE

IN VIVO VALIDATION OF HIGHLY CUSTOMIZED CRANIAL Ti-6AL-4V ELI PROSTHESES FABRICATED THROUGH INCREMENTAL FORMING AND SUPERPLASTIC FORMING: AN OVINE MODEL STUDY.

Brogini et al.

**MATERIALS AND METHODS**

**Microtomography**


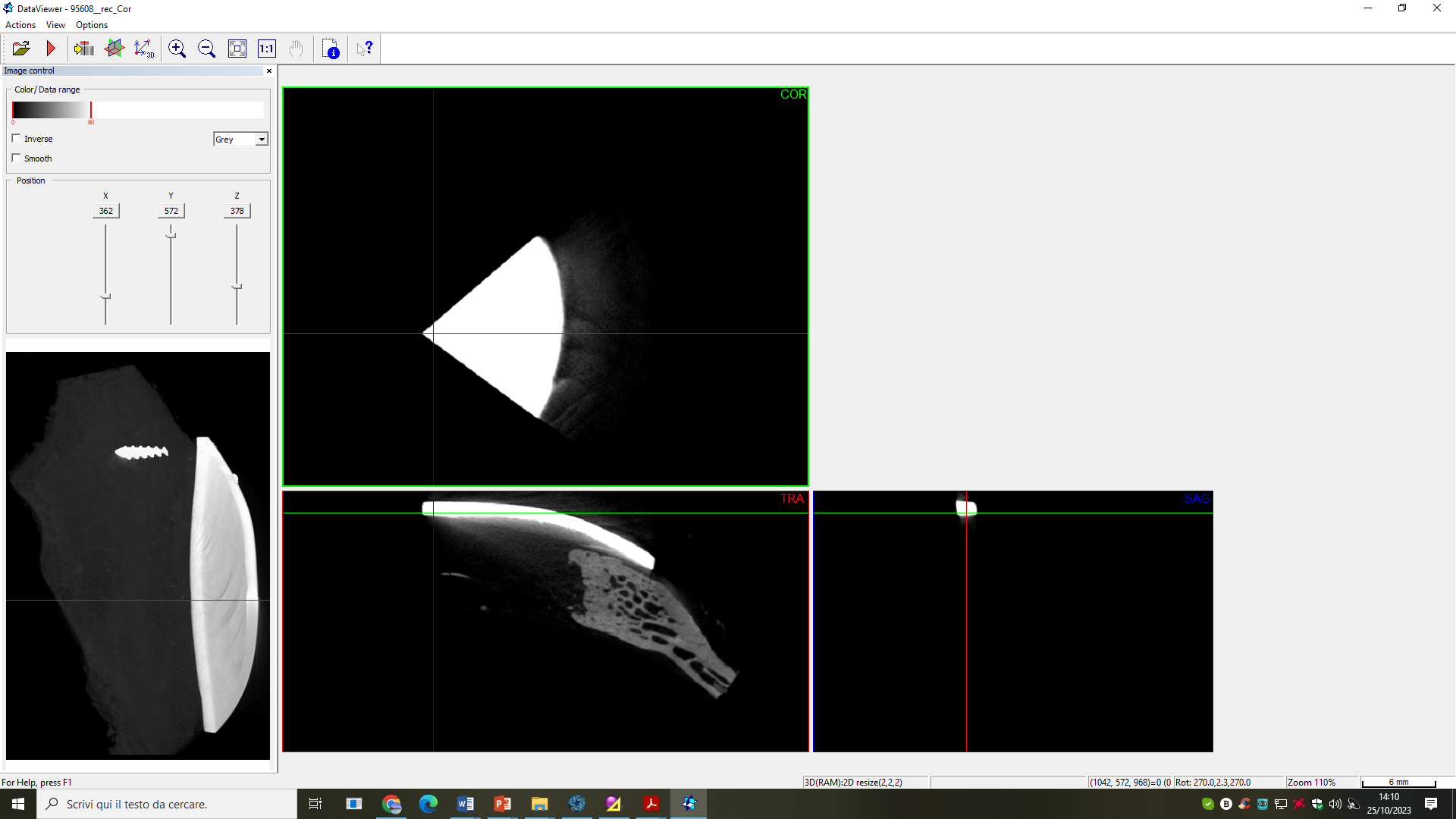


**Figure S1:** Virtual orientation of the reconstructed micro-CT images permits to define a radial cutting plane (TRA) passing between the implant (i.e. defect) center and the bisector of the implant angle inner respect to the skull, as viewed from above (COR). In the image, the worst case sample is reported to better illustrate the concept.


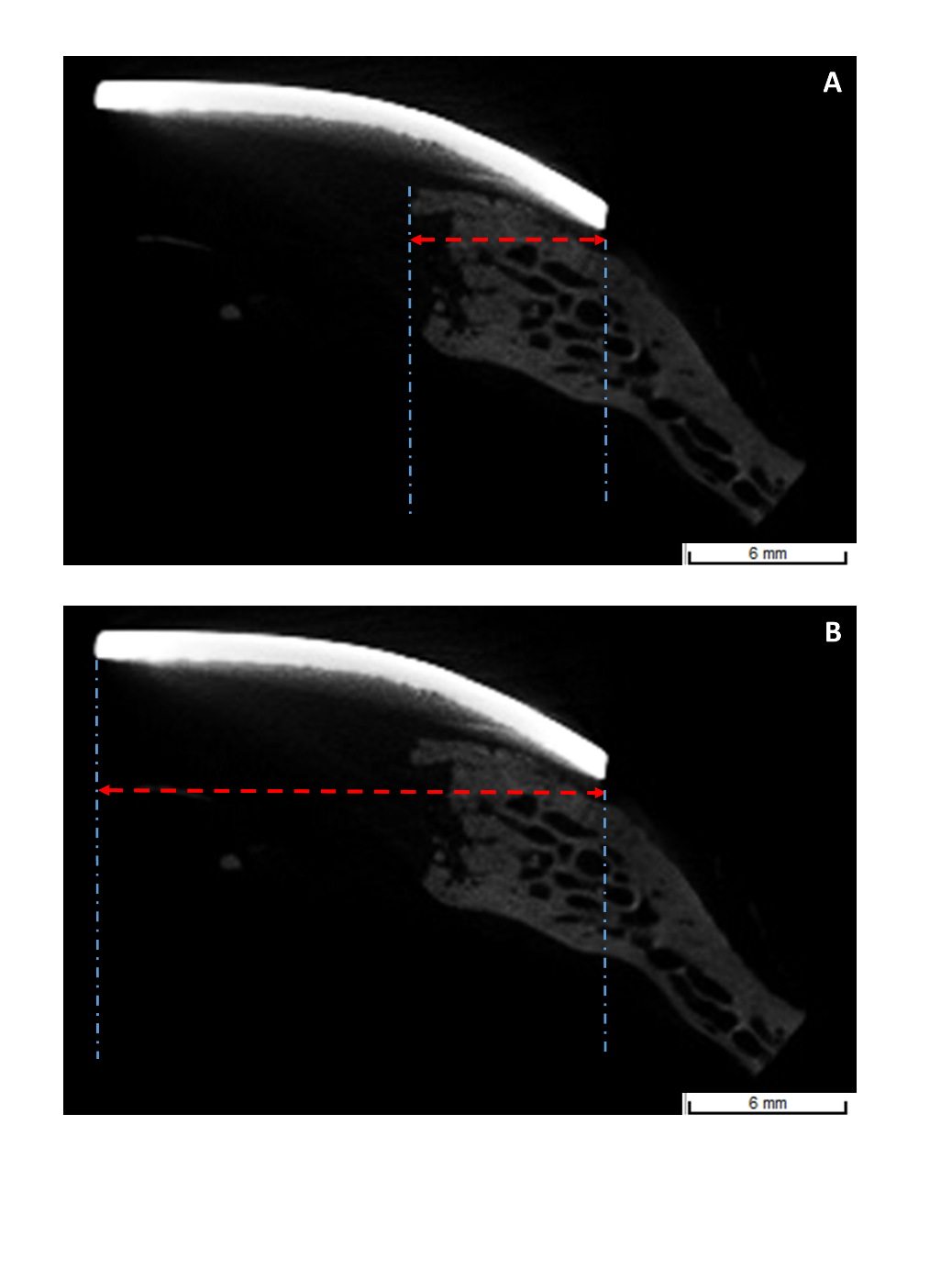


**Figure S2:** In the central micro-CT radial section, the ratio between the line segment connecting the implant's external margin and the margin of the grown bone (A), and the line segment parallel to the previous one and connecting the implant's internal and external margins (B), is defined as defect closure. In the image, the worst case sample is reported to better illustrate the concept.

**RESULTS**

**Microtomography and static histomorphometry**

Figure S3 reports trabecular thickness (Tb.Th), trabecular spacing (Tb.Sp) and trabecular number (Tb.N) measurements. In the considered VOIs, micro-morphometry results for trabecular thickness (Tb.Th), trabecular spacing (Tb.Sp) and trabecular number (Tb.N) showed higher values in SPF compared to SPIF (28%, p < 0.05) and within the SPF group, the 3-month values exceeded those of the 6-month measurements (37%, p < 0.05). Conversely, the parameter Tb.Sp was higher with SPIF prosthesis in comparison to SPF (32%, p < 0.05). Regarding static histomorphometric results, significant effects of ‘type of prosthesis’ factor were highlighted for Tb.Th (Tb.Th: F=20.1, p<0.0005), resulting in significantly higher Tb.Th (18%) values for SPF in comparison to SPIF. A significant lower measurement was observed for Tb.Th (µm) parameters in ROI-2 respect to ROI-1 (Tb.Th: -43%) and ROI-0 (Tb.Th: -31%), for Tb.N (µm) in ROI-2 respect to ROI-1 (-39%) and ROI-0 (-41%), while superior Tb.Sp results (> 100%) were observed in ROI-2 versus ROI-1 and ROI-0.

**
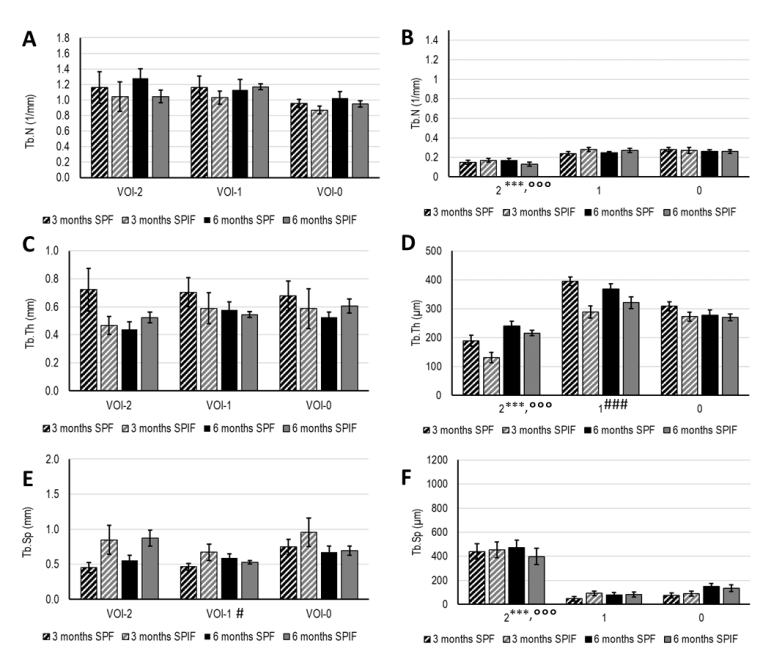
**

**Figure S3:** Microtomography (A, C, E) and histomorphometry (B, D, F) histograms of Tb.N (A, B), Tb.Th (C, D) and Tb.Sp (E, F) reported for experimental times (3 and 6 months), type of prosthesis (SPF and SPIF) and for VOI (VOI-3,-2,-1,-0) or ROI (ROI-2, -1, -0) analyzed. Microtomography LM analysis (Mean ± ES, n=4). Static histmorphometry LMM analysis (Mean ± ES, n=16 repeated measures for each type of prosthesis and experimental time): (ROI effect: Tb.Th: F=125.3, p<0.0005; Tb.N: F=31.7, p<0.0005; Tb.Sp: F=102.5, p<0.0005). One symbol, p<0.05; 2 symbols, p<0.005; 3 symbols, p<0.0005: *, ROI-2 versus ROI-1; °, ROI-2 versus ROI-0; #, VOI-1 (or ROI-1) versus VOI-0 (or ROI-0).

**Dynamic histomorphometry**

**
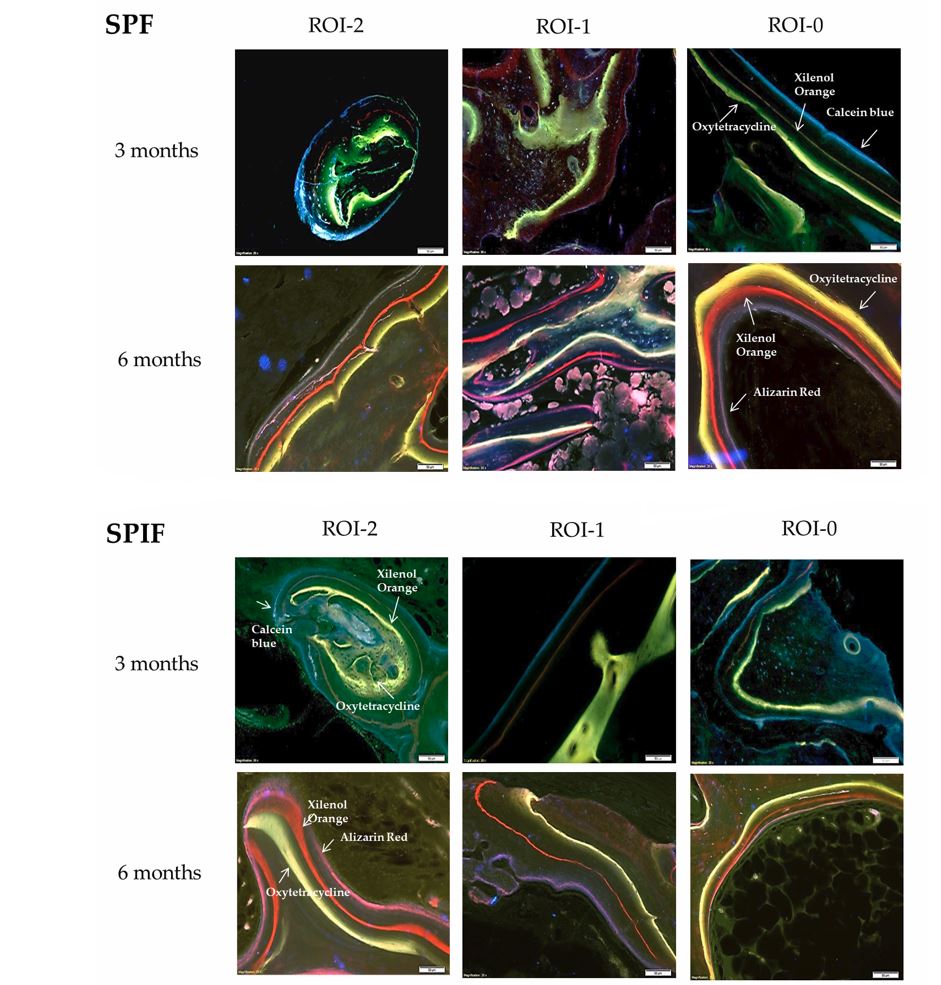
**

**Figure S4:** Representative images of fluorochromes label of SPF and SPIF implants at 3 months and 6 months related to ROIs. For each image the top side is that facing the prosthesis. Scale bar: 50μm

**Histology**

**
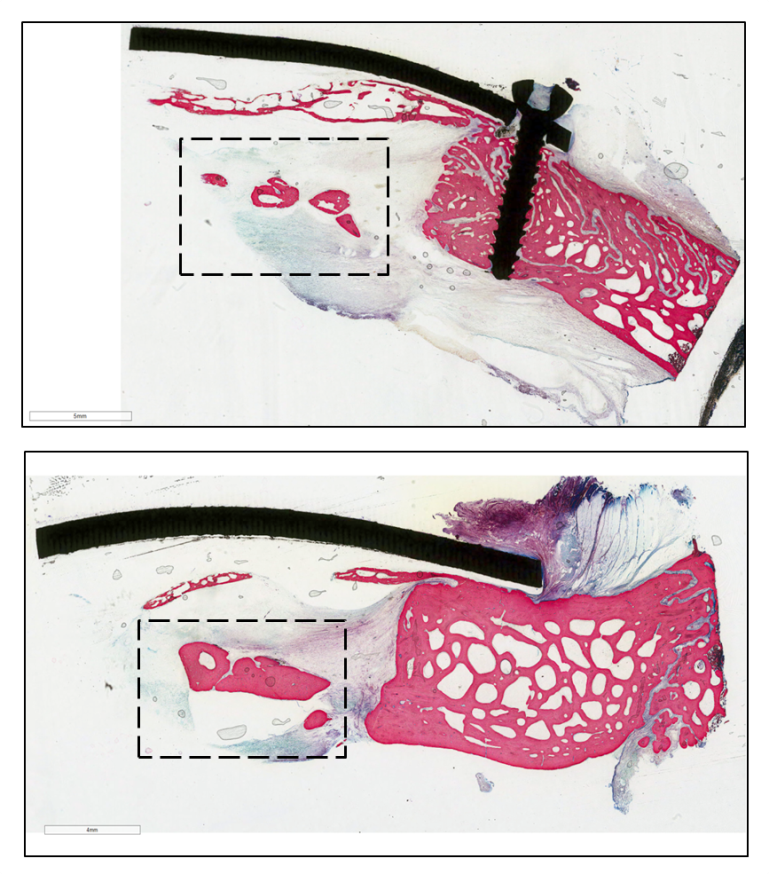
**

**Figure S5:** Representative histological images of new bone formation occurred on the dura side without any contact with the native bone. Stain: Stevenel Blue counterstain with Picrofucsin according to Van Gieson; scale bar: 5mm.
